# Deciphering Femtosecond Charge–Lattice Dynamics in Textured LaFeO_3_ Thin Films

**DOI:** 10.1021/acsnano.6c04204

**Published:** 2026-08-26

**Authors:** Masoud Lazemi, Fabian J. Mohammad, Ryan Ash, Zain Abhari, Roberta Candela, Hans J. F. A. Blankesteijn, Emma van der Minne, Yorick A. Birkhölzer, Miguel Segovia Guzmán, Moritz Nunnenkamp, Phu Tran Phong Le, Sang Han Park, Abhishek Katoch, Soonnam Kwon, Christoph Baeumer, Gertjan Koster, Arnaud Rouzée, Uwe Bergmann, Frank M. F. de Groot

**Affiliations:** † Materials Chemistry and Catalysis, Debye Institute for Nanomaterials Science, 3230 Utrecht University, Universiteitsweg 99, 3584 CG Utrecht, The Netherlands; ‡ MESA+ Institute for Nanotechnology, University of Twente, P.O. Box 217, Enschede 7500 AE, The Netherlands; § Max Born Institute for Nonlinear Optics and Short Pulse Spectroscopy, Max-Born-Straße 2A, 12489 Berlin, Germany; ∥ Department of Physics, University of Wisconsin–Madison, Madison, Wisconsin 53706-1390, United States; ⊥ 92226 Pohang Accelerator Laboratory, Pohang, Gyeongbuk 37673, Republic of Korea; # Department of Chemistry, 26721 Yonsei University, Seoul 03722, Republic of Korea

**Keywords:** extreme-ultraviolet (XUV) absorption spectroscopy, Fe 3p edge, textured LaFeO_3_ thin films, ligand-to-metal charge-transfer (LMCT), pulsed laser deposition, Ca_2_Nb_3_O_10_ nanosheets, multiplet calculations

## Abstract

Understanding the intricate interplay between electronic and lattice dynamics is indispensable for controlling photoinduced functionality in correlated oxides. Here, we investigate the ultrafast relaxation pathways in textured LaFeO_3_ using femtosecond extreme-ultraviolet (XUV) absorption spectroscopy at the iron 3*p* edge. These nanosheets facilitate oriented perovskite growth on the amorphous Si_3_N_4_ membrane, giving rise to local epitaxy while preserving XUV transparency. By combining table-top time-resolved measurements with crystal-field multiplet calculations, we track the evolution from rapid ligand-to-metal charge transfer (LMCT) excitation to a lattice-stabilized polaronic state. The early-time response is dominated by the population of a mixed charge-transfer state with pronounced t_2g_^3^e_g_^3^ (^5^ *E*) character, which indicates the initial electronic redistribution that follows photoexcitation. On subpicosecond time scales, the emergence of lattice-assisted carrier localization leads to partial charge localization and a lowering of the symmetry of Fe^3+^ sites. These changes suggest the formation of a polaronic state. At later delays, the transient spectra are governed by distorted Fe^3+^ configurations with residual charge-transfer character, without evidence for long-lived Fe^2+^ formation. Comparison with α-Fe_2_O_3_ reveals distinct material dependence in both charge-transfer relaxation and polaron-formation dynamics, demonstrating the roles of lattice topology and iron–oxygen covalency.

## Graphical abstract

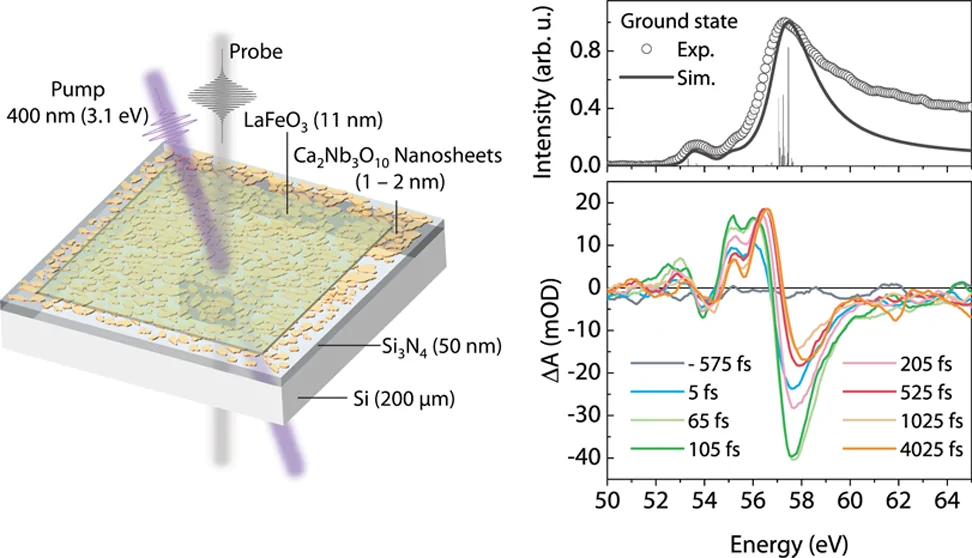

## 1 Introduction

Ultrafast optical excitation drives correlated transition-metal oxides into nonequilibrium electronic states in which charge redistribution, local symmetry changes, and lattice motion are inextricably intertwined on femtosecond-to-picosecond time scales.^1–3^ Thus, deciphering how these coupled processes evolve and how an initially delocalized excitation relaxes toward a stabilized configuration is indispensable for elucidating photoinduced phenomena such as phase transitions, charge transport, and magnetic reordering.^4–6^ Despite remarkable experimental and theoretical efforts, the sequence of electronic and structural events following photoexcitation remains incompletely resolved for many prototypical oxide systems.

Lanthanum iron oxide (LaFeO_3_) is a particularly suitable model material for addressing this problem. It is a perovskite antiferromagnetic charge-transfer insulator with strong iron–oxygen covalency, in which the corner-sharing FeO_6_ octahedral network enables efficient coupling between electronic excitations and lattice degrees of freedom.^7^ Optical excitation across the charge-transfer gap promotes electrons from oxygen-derived states into the iron 3*d* state, generating ligand-to-metal charge-transfer (LMCT) configurations that are intrinsically unstable with respect to lattice relaxation.^8–10^ Whether and how such excitations localize and on what time scale structural reorganization accompanies this process remain central open questions.

Femtosecond X-ray absorption spectroscopy (fs-XAS) has emerged as a powerful experimental tool for tracking these processes with element specificity.^11–21^ We recently demonstrated that fs-XAS studies at the oxygen 1*s* (O K-edge; 1*s* → 2*p* transition) and iron 2*p* (Fe L_2,3_-edges; 2*p* → 3*d* transition) of LaFeO_3_ lead to rapid LMCT formation followed by lattice-coupled relaxation on subpicosecond to picosecond time scales.^11^ While these measurements have established a broad phenomenological picture, their interpretation is strongly influenced by core-hole effects and, at the 2*p* edge, by significant spin–orbit coupling.^22,23^ As a result, early-time symmetry changes and the initial stages of charge localization are difficult to separate from later structural responses. In that study, the oxygen 1*s* and iron 2*p* edges identified the formation of photoinduced polarons in LaFeO_3_. In contrast, the present investigation of the iron 3*p* edge is particularly sensitive to local iron-site symmetry and exchange interactions, which reveal the earliest stages of charge localization and lattice-coupled relaxation.

Time-resolved spectroscopy at the iron 3*p* edge (M_2,3_-edge; 3*p* → 3*d* transition) provides a complementary perspective. The reduced spin–orbit coupling of the 3*p* core level, together with strong 3*p*–3*d* exchange interactions, makes iron 3*p* X-ray absorption particularly sensitive to local electronic configuration, spin multiplicity, and deviations from ideal octahedral symmetry.^23,24^ Relatively small changes in orbital occupancy or crystal-field splitting can therefore lead to pronounced modifications of the multiplet structure at this edge. These characteristics make femtosecond iron 3*p* spectroscopy well suited to probing the earliest stages of charge localization and lattice-assisted relaxation. Nevertheless, experimental realizations remain challenging due to the need for ultrashort extreme-ultraviolet (XUV) pulses and stringent sample-preparation requirements.

From a theoretical standpoint, interpreting iron 2*p* and 3*p* X-ray absorption spectra in correlated systems requires approaches that explicitly account for strong electron–electron interactions and multiplet effects.^23^ Crystal-field multiplet calculations offer a robust framework for interpreting both 2*p* and 3*p* XAS in such materials,^9,23,25–28^ whereas conventional approaches such as density functional theory (DFT) or single-particle models do not adequately capture the many-body interactions and multiplet splitting that dominate the spectral features at these edges.^9,23,28^ As a consequence, multiplet-based methods are imperative for disentangling electronic configuration changes from lattice-induced symmetry lowering in time-resolved core-level spectroscopy.

In this work, we employ femtosecond XUV absorption spectroscopy at the iron 3*p* edge to investigate the ultrafast photoinduced response of textured LaFeO_3_ thin films. We have prepared these samples on Ca_2_Nb_3_O_10_ nanosheet-templated Si_3_N_4_ membranes, enabling transmission-mode measurements while preserving the intrinsic perovskite lattice environment. By combining time-resolved spectroscopy with crystal-field multiplet analysis, this approach serves as a stepping stone toward elucidating how LMCT excitation, lattice-assisted charge localization, and local symmetry changes evolve on ultrafast time scales, and how these processes depend on lattice topology.

## 2 Results and Discussions

### 2.1 Transmission-Mode XUV Measurements and Sample Architecture

Figure 1 depicts the experimental setup used for the pump–probe XUV experiment on textured LaFeO_3_ thin films carried out at the X-FAST instrument^29^ (see Methods section). These kinds of experiments on solid-state samples at XUV photon energies in transmission mode face experimental challenges related to the short penetration depth of the XUV probe beam. To ensure sufficient transmission, one needs to employ very thin samples and substrates on the order of several nm. Compared to gas phase experiments,^26^ the 50 nm Si_3_N_4_ sample substrate alone absorbs a substantial portion of the XUV photons, reducing the flux to approximately 10% with an additional loss of similar magnitude from the sample.

**Figure 1:**
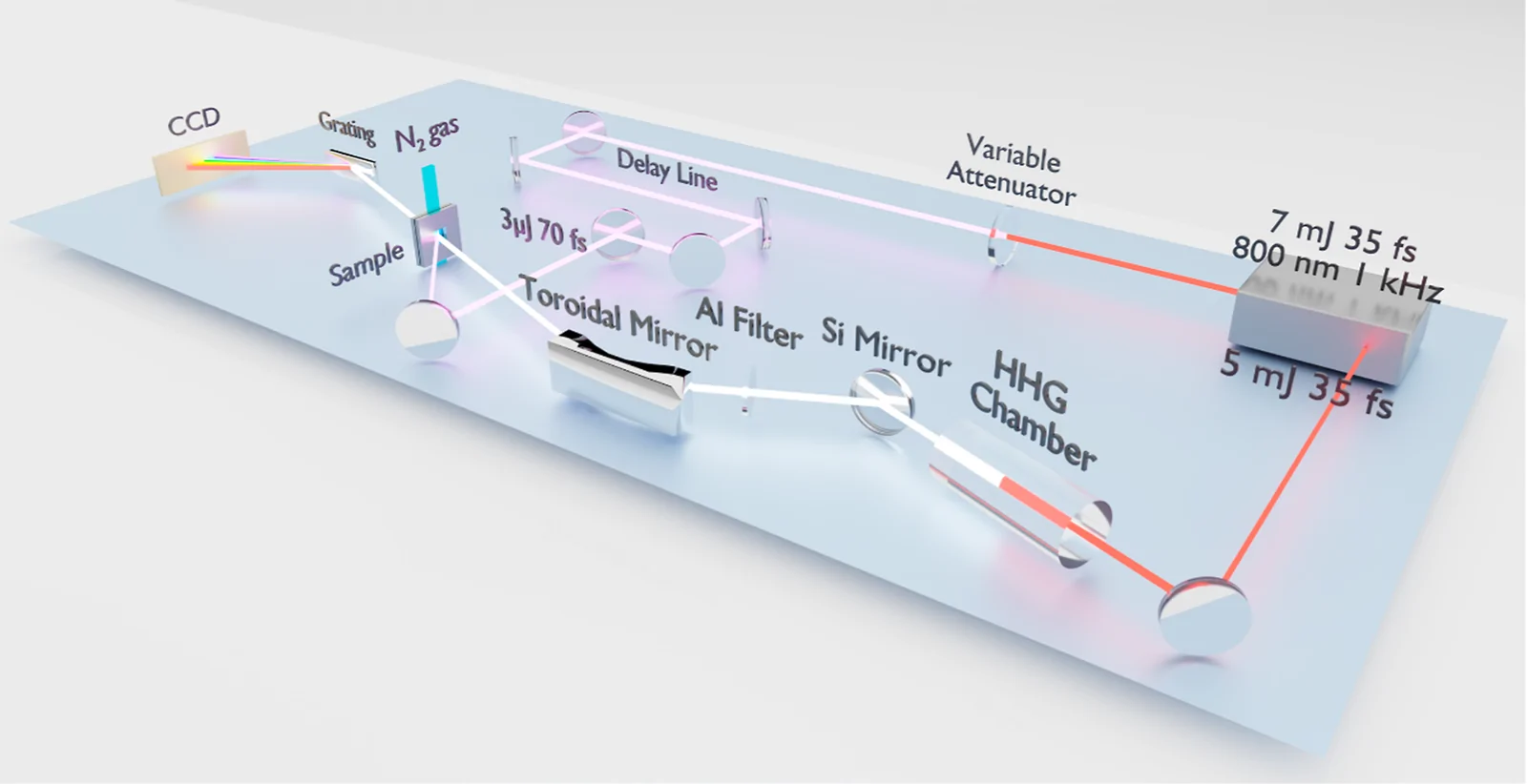
Schematic of pump–probe setup for XUV experiment using the X-FAST instrument.^29^ A single Ti/sapphire laser generates both the XUV and pump beams, which are separated inside the laser amplifier using a polarizing beam splitter. A 5 mJ portion of the laser output is directed toward high-harmonic generation (HHG) in a semi-infinite gas cell with Ne as the generating medium. The resulting XUV pulse is isolated using a Si mirror and an Al filter and subsequently focused onto the sample with a toroidal mirror. The remaining laser output is attenuated, passed through a variable delay line, and focused onto the sample. The sample was excited with a 3 μJ, 70 fs pulse at 400 nm. A stream of N_2_ gas was directed across the sample inside a gas-cooling cell to facilitate heat dissipation and avoid beam damage. Finally, a custom-built spectrometer, incorporating a variable line-space grating and a charge-coupled device (CCD) detector, records the XUV spectrum.^29^

Studying thermally-sensitive chemical and condensed matter systems, particularly those undergoing thermal decomposition, thermally-induced phase transitions, or other temperature-dependent dynamics, causes additional experimental challenges. This difficulty arises from the limited heat dissipation in thin-film samples. We have mitigated this issue by directing a room-temperature stream of *N*_2_ gas across the sample inside a gas-cooling cell at the X-FAST instrument,^29^ as depicted in Figure 1. The setup consists of two silicon nitride windows—one with the sample deposited on the side facing the gas flow and the other a pristine membrane—secured with face plates against the cell. This configuration resulted in an overall transmission rate of 30%.

It should be mentioned that the amorphous Si_3_N_4_ membranes inherently prevent the direct growth of perovskite films, thereby requiring a novel approach to enable oriented oxide growth. Herein, we have employed Ca_2_Nb_3_O_10_ nanosheets^30^ as a seed layer, representing a viable strategy for the growth of high-quality textured LaFeO_3_ thin films on amorphous Si_3_N_4_ membranes, where direct perovskite growth is otherwise not feasible (Figure 2a). The detailed procedure for Ca_2_Nb_3_O_10_ deposition using the Langmuir–Blodgett technique is described in the Supporting Information (Figure S1).

**Figure 2:**
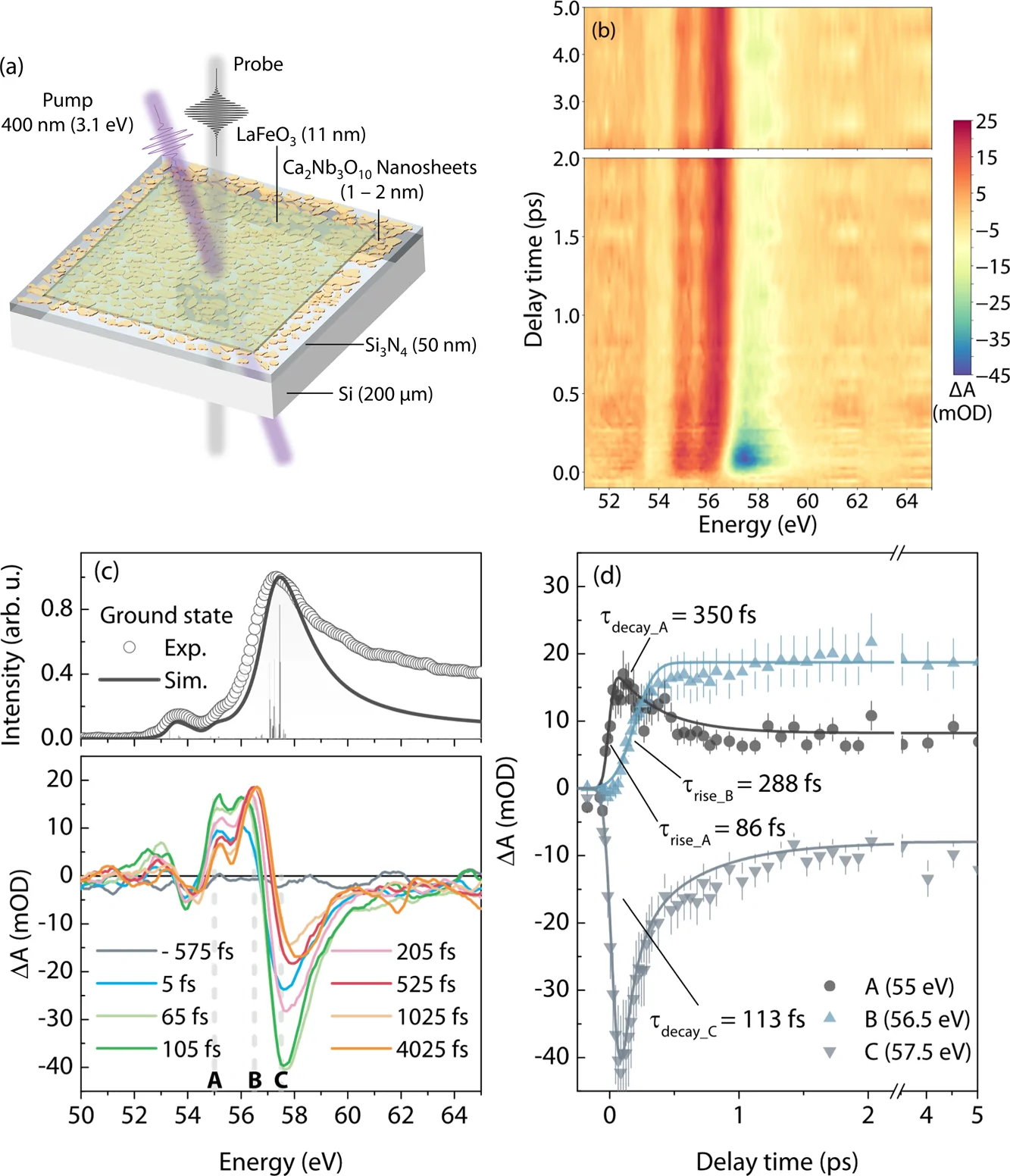
Sample architecture, static and time-resolved iron 3*p* spectra. (a) Schematic illustration of the textured LaFeO_3_ thin film grown on a Ca_2_Nb_3_O_10_ nanosheet-templated Si_3_N_4_ membrane. The nanosheet template promotes oriented perovskite growth on the amorphous membrane while preserving XUV transparency. (b) The transient spectral changes following 400 nm photoexcitation, with the differential absorption (Δ*A* = −log­(*I*_pump_/*I*_nopump_)) plotted as a function of pump–probe delay. The differential absorption shows regions of increased (red) and decreased (blue) absorption near 56 and 57 eV. The data is visualized using 120 contour levels. (c) Top panel: experimental and simulated ground-state 3*p* XAS. The vertical sticks indicate crystal field multiplet transitions. The calculation parameters are reported in Table S1. The background subtraction procedure is discussed in the Supporting Information (c) Bottom panel: Transient spectra at different delay times. The spectra were smoothed using a 5-point moving average applied after averaging over a 30 fs time window. (d) Kinetic traces extracted at features A (55.0 eV), B (56.5 eV), and C (57.5 eV) using an energy window of 400 meV. Vertical lines depict the error bars. Solid lines correspond to fits of the experimental data using eq S2. The detailed fittings and kinetic constants obtained for feature (A–C) are reported in Figures S10–S12 and Tables S2–S4.

Figure 2a schematically illustrates the architecture of the textured LaFeO_3_ thin film prepared on a Ca_2_Nb_3_O_10_ nanosheet-templated Si_3_N_4_ membrane, which enables transmission-mode XUV at the iron 3*p* edge. The use of an ultrathin, XUV-transparent Si_3_N_4_ membrane results in minimal absorption background while maintaining sufficient mechanical stability for pump–probe measurements. A monolayer of Ca_2_Nb_3_O_10_ nanosheets is first deposited onto the amorphous Si_3_N_4_ membrane, serving as a crystalline seed layer that promotes oriented growth of the LaFeO_3_ film. Although the underlying membrane lacks long-range order, the nanosheet template imposes local structural order, enabling the subsequent deposition of a textured LaFeO_3_ perovskite layer. This approach circumvents the intrinsic incompatibility between amorphous membrane substrates and epitaxial oxide growth.

The resulting LaFeO_3_ thin film exhibits preferential orientation normal to the membrane surface, as confirmed by the structural characterization presented in the Supporting Information (Figures S2 and S3). The film thickness is chosen to balance sufficient X-ray absorption contrast at the iron 3*p* edge with high transmission in the extreme-ultraviolet spectral range, while also yielding optimal signal-to-noise in time-resolved measurements. This nanosheet-templated membrane platform is of paramount importance for accessing ultrafast iron 3*p* absorption in transmission geometry, which would otherwise be impractical for complex oxides. By combining structural templating with XUV transparency, the sample design shown in Figure 2a enables element-specific probing of electronic and lattice dynamics in perovskite oxides on femtosecond time scales.

The sample was excited by a 400 nm (3.1 eV) optical pump laser, which was above the band gap of LaFeO_3_ (≈2.0 eV).^7,11^ The spot sizes of the pump and probe beams were approximately 200 and 80 μm, respectively. Given that the nanosheets exhibit lateral dimensions ranging from 1 to 5 μm (Figure S2), the measurements reflect collective, ensemble-averaged properties rather than responses from individual nanosheets.

### 2.2 Ground-State Iron 3*p* X-ray Absorption

The top panel of Figure 2c shows the ground-state (GS) iron 3*p* XAS spectrum of LaFeO_3_ measured before optical excitation. The overall line shape and multiplet structure are in strong agreement with previously reported iron 3*p* spectra of octahedrally coordinated Fe^3+^ compounds, such as hematite (α-Fe_2_O_3_), confirming the high-spin Fe^3+^ character of the as-prepared film.^22,26,27^ Hence, the spectrum demonstrates a reliable ground-state reference for subsequent time-resolved measurements.

Iron 3*p* XAS involves the excitation of a 3*d* ^*n*^ ground-state configuration into a 3*p* ^5^3*d* ^*n*+1^ final state, in which strong core-hole interactions and 3*p*–3*d* exchange coupling play a principal role in shaping the spectral features.^22,23^ For Fe^3+^ in LaFeO_3_, the ground-state electronic configuration corresponds to a high-spin 3*d* ^5^ state, t_2g↑_^3^e_g↑_^2^, associated with the ^6^ *A*_1g_ term, where all five 3*d* electrons are aligned in the same spin channel.^11^ The resulting multiplet structure captures the combined influence of crystal-field splitting, exchange interaction, and covalent hybridization with oxygen ligands.

At the iron 3*p* edge, the absorption process probes transitions from the iron 3*p* core level into unoccupied states with iron 3*d* character. In LaFeO_3_, it has already been shown that the iron 3*d* states are strongly hybridized with oxygen 2*p* orbitals.^7,11^ As a consequence, the unoccupied states probed in the iron 3*p* XAS experiment are not purely metal-centered but also contain a significant ligand contribution. The resulting ground-state spectrum therefore constitutes not only the Fe^3+^ multiplet structure but also the degree of iron–oxygen covalency, which plays a central role in determining the sensitivity of the iron 3*p* edge to changes in electronic configuration and local symmetry.

Upon optical excitation at 400 nm, an electron is promoted from oxygen 2*p* states into the iron 3*d* shell, forming an LMCT configuration that can be described as t_2g↑_^3^e_g↑_^2^ + t_2*g*↓_^1^ + *L* , where *L* denotes a ligand hole arising from iron–oxygen hybridization. In particular, the optical excitation inverts the parity of the electronic state. Starting from an odd-parity 3*d* ^5^ ground state, the optically excited configuration corresponds to an even-parity 3*d* ^6^ *L* state. By contrast, the 3*d* ^6^ *L* configurations admixed into the 3*d* ^5^ ground state arise through iron–oxygen covalency and therefore inherit the overall odd parity of the ground-state wave function. Consequently, the photoexcited even-parity 3*d* ^6^ *L* state is symmetry-distinct from the odd-parity 3*d* ^6^ *L* configurations present in the ground state. Although both states share the same nominal 3*d* ^6^ *L* electronic occupation, they differ in their symmetry character and therefore represent distinct quantum states. As a result, the photoexcitation does not merely enhance the weight of the covalently admixed 3*d* ^6^ *L* configurations already present in the ground state, but it generates a distinct excited-state 3*d* ^6^ *L* configuration with different symmetry properties.^11^

This distinction has significant consequences for the interpretation of the transient iron 3*p* spectra. As the ground-state absorption intensity originates from parity mixing between odd 3*d* ^5^ and odd 3*d* ^6^ *L* configurations, the photoexcited even 3*d* ^6^ L state does not participate in the same mixing channel. As a result, photoexcitation leads to a redistribution of spectral weight that cannot be described as a simple population shift within the ground-state configuration. Instead, changes in the iron 3*p* absorption constitute the formation of new electronic configurations with different parities and exchange couplings, providing a sensitive handle on LMCT and its subsequent evolution. The ground-state spectrum shown in the top panel of Figure 2c thus defines both the electronic reference and the symmetry framework required for interpreting the transient response. Deviations from this spectrum at finite pump–probe delays can be directly attributed to the population of parity-distinct LMCT states and, at later times, to additional symmetry-lowering induced by lattice reorganization.

### 2.3 Early-Time Dynamics (5–100 fs)

Figure 2b,c depict the transient iron 3*p* absorption spectra of LaFeO_3_ following 400 nm photoexcitation. Within the experimental time resolution, evident spectral changes emerge across the M_2,3_ edge, consisting of two positive absorption features centered at approximately 55.0 eV (feature A) and 56.5 eV (feature B), accompanied by a bleach at 57.5 eV (feature C). The simultaneous onset of these features indicates that the prevailing electronic excitation pathway emerges impulsively after photoexcitation.

The ground-state iron 3*p* spectrum shown in the top panel of Figure 2c exhibits the characteristic multiplet structure of high-spin Fe^3+^ in an octahedral ligand field, attesting strong 3*p*–3*d* exchange interactions. Upon excitation, the redistribution of spectral weight observed in Figure 2b extends beyond simple depopulation of the ground state and requires population of excited electronic configurations with distinct multiplet fingerprints. Therefore, the early-time transient line shape reveals the nature of the photoexcited electronic state.

To identify the origin of this response, we compared the experimental spectra in the 5–100 fs window with crystal-field multiplet calculations for candidate excited states. As shown in Figure 3a, the transient spectra can be reproduced by a linear combination of LMCT configurations corresponding to the t_2g_^3^e_g_^3^ (^5^ *E*) and t_2g_^4^e_g_^2^ (^5^ *T*_2_), with relative weights of approximately 80% (^5^ *E*) and 20% (^5^ *T*_2_). The calculated spectra for these states and the ground-state reference are shown in Figure S5, which demonstrate distinct energy positions and relative intensities that closely match the experimentally observed features A–C.

**Figure 3:**
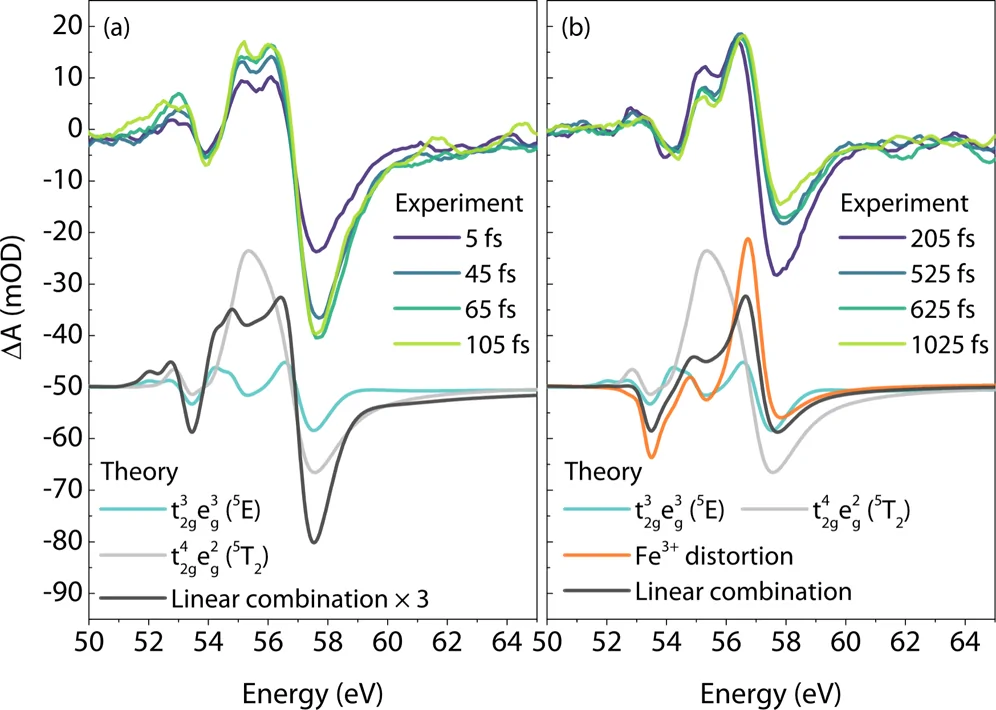
Ultrafast electronic evolution from LMCT excitation to lattice-coupled states. Time-resolved iron 3*p* spectra reveal the evolution from LMCT excitation to lattice-coupled electronic states on femtosecond time scales. (a) Transient iron 3*p* spectra between 5 and 100 fs following 400 nm (3.1 eV) excitation, compared with calculated spectra for the t_2g_^3^e_g_^3^ (^5^ *E*) and t_2g_^4^e_g_^2^ (^5^ *T*_2_) configurations, as well as their linear combination of 80% (^5^ *E*) and 20% (^5^ *T*_2_). (b) Transient spectra between 200 fs and 1 ps compared with calculated spectra for t_2g_^3^e_g_^3^ (^5^ *E*), t_2g_^4^e_g_^2^ (^5^ *T*_2_), and Fe^3+^ distortions, together with their linear combination of 20%, 25%, and 55%, respectively. The Fe^3+^ distortion spectrum was calculated considering *D*_s_ = *D*_t_ = 0.2 eV, as detailed in Supporting Information (Figure S7).

The ascendancy of the (^5^ *E*) contribution manifests that the primary photoexcitation involves promotion of an electron from oxygen-derived states into iron 3*d* orbitals with substantial e_g_ character, consistent with optical excitation across the charge-transfer gap.^8,23^ The finite admixture of the (^5^ *T*_2_) configuration, however, is indispensable to account for the detailed line shape, particularly the relative intensities of features A and B. This implies that the early-time excited state is best described as a well-defined superposition within the iron–oxygen hybridized electronic state instead of a single LMCT eigenstate.

Several alternative excitation scenarios can be excluded on firm spectroscopic grounds, consistent with prior analyses of iron oxide multiplet fingerprints at the M-edge.^9,23,31^ Calculated spectra for low-spin and intermediate-spin iron configurations (Figure S6) fail to reproduce both the energy separation and sign pattern of the transient features, ruling out ultrafast spin-state crossover as the dominant mechanism. Likewise, pure *d*–*d* excitations within the iron 3*d* shell (Figure S8) do not yield the characteristic positive–negative feature sequence observed experimentally, and metal–metal charge-transfer (MMCT) configurations (Figure S9) exhibit spectral signatures that are incompatible with the measured transient response. Consequently, these comparisons identify the LMCT as the origin of the early-time dynamics.

The kinetic traces extracted at features A, B, and C (Figure 2d) further support this assignment. All three features rise within the instrument response function, confirming that LMCT excitation occurs on a time scale shorter than the experimental resolution. Detailed fitting of the kinetic traces (Figures S10–S12 and Tables S2–S4) shows that introducing additional exponential components does not yield statistically significant improvement, attesting that the early-time population of the LMCT state is well described by a single prompt excitation process. Feature C, corresponding to the ground-state bleach, exhibits an opposite sign but follows a similar temporal evolution (Figures 2d and S12), consistent with rapid redistribution of population out of the Fe^3+^ ground state.^26^

Notably, during the first 100 fs, the transient spectra evolve primarily in amplitude rather than in energy position or line-shape complexity (Figure 3a). This behavior affirms that, at these earliest delays, the local FeO_6_ geometry remains intact mainly. Hence, the lattice motion has not yet made a considerable contribution to the spectral response. The iron 3*p* edge, with its reduced spin–orbit coupling and strong exchange sensitivity, therefore probes a regime dominated by electronic reconfiguration within an essentially frozen lattice. This early-time LMCT excitation initiates the sequence of subsequent relaxation processes. While no spectroscopic signatures of lattice-assisted charge localization are evident within the first 100 fs, the presence of mixed LMCT character already establishes the conditions under which electron–phonon coupling can act. As discussed below, the redistribution of spectral weight away from the (^5^ *E*)-dominated state at later times marks the onset of lattice involvement and the formation of a polaronic excited state.^27^

### 2.4 Intermediate-Time Regime (200 fs–1 ps)

Following the impulsive population of LMCT-derived electronic configurations, the transient iron 3*p* spectra of LaFeO_3_ evolve on subpicosecond time scales, unraveling the increasing involvement of lattice degrees of freedom. As shown in Figure 2c (bottom panel), between approximately 200 fs and 1 ps, the spectral response no longer changes solely in magnitude. Instead, it exhibits a systematic redistribution of intensity, accompanied by modifications to the multiplet line shape. This regime is a hallmark of the transition from a predominantly electronic excitation toward a lattice-coupled, partially localized excited state.

The evolution of features A and B shows a clear spectroscopic signature of this transition. Feature A, associated primarily with LMCT-derived multiplet intensity, decays on a characteristic subpicosecond time scale, while feature B gains relative weight and broadens. This behavior is discernible in the kinetic traces in Figure 2d and requires an additional contribution beyond relaxation within a fixed LMCT state. This contribution is linked to local symmetry breaking at the iron site.

Crystal-field multiplet analysis of the spectra in this time window corroborates this interpretation. As shown in Figure 3b, the transient spectra between 200 fs and 1 ps are reproduced by a linear combination of t_2g_^3^e_g_^3^ (^5^ *E*), t_2g_^4^e_g_^2^ (^5^ *T*_2_), and Fe^3+^ distortions, with relative weights of approximately 20%, 25%, and 55%, respectively. Compared with the early-time response, in which LMCT-derived states prevail, the appearance of a considerable distorted Fe^3+^ contribution represents a qualitative change in the nature of the excited state.

The distorted Fe^3+^ configurations are modeled using finite tetragonal crystal-field parameters, as detailed in Figure S7. These parameters serve as effective descriptors of local symmetry lowering and capture the sensitivity of the iron 3*p* multiplet structure to lattice reorganization. The progressive increase of the distorted Fe^3+^ contribution, besides the concomitant reduction of LMCT weight, implies that electronic excitation and lattice relaxation proceed concurrently during this intermediate stage.

#### 2.4.1 Material-Dependent Charge-Transfer Relaxation: LaFeO_3_ versus α-Fe_2_O_3_

A comparison with corundum-structured α-Fe_2_O_3_ reveals that the relaxation dynamics observed here are strongly material-dependent. Using a probe energy closely matching that employed by Vura-Weis et al.,^26^ we find that the LMCT state in LaFeO_3_ decays with a characteristic time constant of approximately 350 fs (Figures 2d and S10), which is significantly slower than in α-Fe_2_O_3_. Applying the same fitting procedure described here (eq S2) to the data reported by Vura-Weiss et al.^26^ and Carneiro et al.^27^ yields an initial, fast decay of the CT feature, with a time constant of approximately 185 fs in both data sets. The slower decay of 350 fs in LaFeO_3_ implies a longer stabilization of the charge-transfer configuration within the perovskite lattice, which indicates stronger iron–oxygen covalency and enhanced electronic delocalization, supported by the corner-sharing FeO_6_ network.

In α-Fe_2_O_3_, by contrast, the different octahedral connectivity and greater lattice rigidity promote more rapid charge-transfer relaxation. The faster decay observed in the corundum structure indicates that electronic localization is less effectively stabilized at early times, leading to a shorter-lived LMCT state. The comparison thus demonstrates that the lifetime of the charge-transfer configuration is not universal across iron oxides, but is instead governed by lattice topology and bonding characteristics.

#### 2.4.2 Polaron Formation Dynamics

The same energy-consistent comparison allows a quantitative assessment of polaron formation dynamics. When probed at comparable energies, polaron formation in LaFeO_3_ is found to occur approximately 100 fs faster than in α-Fe_2_O_3_.^26^ This accelerated onset reveals the more efficient lattice reorganization enabled by the perovskite structure, in which cooperative distortions of the corner-sharing FeO_6_ octahedra can develop rapidly once charge localization begins. The difference in polaron formation times, therefore, stems from intrinsic lattice dynamics.

In fact, the coexistence of residual LMCT-derived character with a growing distorted Fe^3+^ contribution in Figure 3b further emphasizes this picture. Charge localization proceeds progressively, with the electronic excitation retaining a hybridized iron–oxygen character while the lattice adapts to stabilize the localized state. The absence of spectral signatures associated with Fe^2+^ configurations, as evidenced by the exclusion analyses in Figures S6–S9, endorses that this process involves lattice-assisted localization without long-lived reduction of the iron center.

It should be noted that additional insight can be obtained by comparing the polaron formation times extracted from iron 3*p* vs 2*p* fs-XAS measurements of LaFeO_3_. In our recent iron 2*p* study,^11^ a characteristic polaron formation time of approximately 600 fs was reported, nearly a factor of two longer than the ≈300 fs extracted here from the iron 3*p* data. This difference unveils the distinct sensitivities of the two absorption edges.

The iron 2*p* edge is dominated by strong spin–orbit coupling and pronounced core-hole effects, which emphasize changes in oxidation state and later stages of lattice relaxation. The iron 3*p* edge, in contrast, exhibits reduced spin–orbit coupling and enhanced sensitivity to exchange interactions and local symmetry changes. As a consequence, the 3*p* edge captures the onset of polaronic distortion at an earlier stage of the relaxation process, yielding a shorter apparent formation time. Hence, these two probes interrogate complementary perspectives on the same underlying dynamics. Whereas iron 2*p* fs-XAS predominantly tracks the development of the fully formed polaronic state through its sensitivity to oxidation-state changes and lattice relaxation, iron 3*p* is particularly sensitive to local symmetry breaking and exchange interactions, which enables the observation of the initial charge–lattice coupling processes that precede complete polaron formation.

### 2.5 Late-Time Regime (3–5 ps)

At longer pump–probe delays, the transient iron 3*p* spectra of LaFeO_3_ evolve into a quasi-stationary line shape that persists throughout the measured time window. As shown in Figure 4, the spectra at 3, 4, and 5 ps are nearly identical, indicating that the dominant relaxation processes have already concluded and that the system has reached a stabilized excited-state configuration. This late-time response sheds light on the nature of the long-lived photoinduced state.

**Figure 4:**
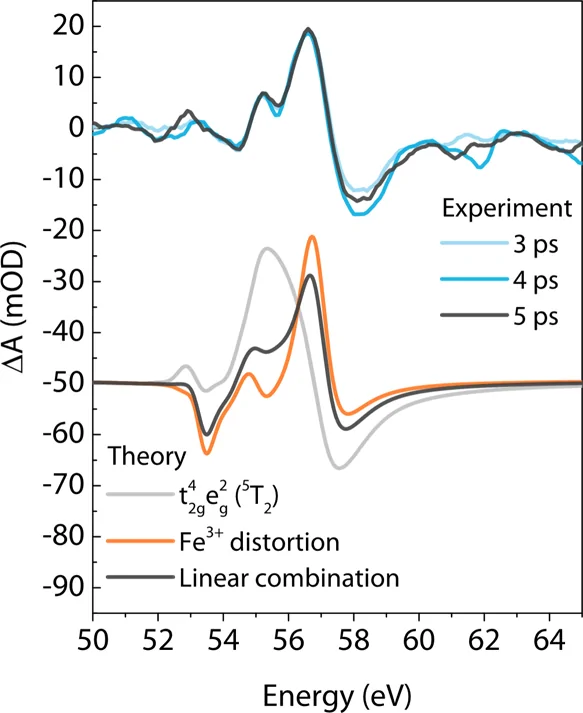
Lattice-stabilized state at picosecond delay times. At picosecond delay times, the transient iron 3*p* spectra converge to a lattice-stabilized configuration. Transient iron 3*p* spectra at pump–probe delays of 3, 4, and 5 ps following 400 nm excitation, compared with calculated spectra for the t_2g_^4^e_g_^2^ (^5^ *T*_2_) configuration and Fe^3+^ distortions. The experimental spectra are reproduced by a linear combination of 30% (^5^ *T*_2_) and 70% distorted Fe^3+^ character, indicating dominant lattice stabilization with residual charge-transfer contribution.

Quantitative comparison with crystal-field multiplet calculations reveals that the spectra in this regime are reproduced by a linear combination of t_2g_^4^e_g_^2^ (^5^ *T*_2_) and Fe^3+^ distortions, with relative weights of approximately 30% and 70%, respectively. The predominance of the distorted Fe^3+^ contribution signifies that lattice reorganization now governs the spectral response. At the same time, the persistence of a finite (^5^ *T*_2_) component signifies that the electronic excitation retains a measurable degree of LMCT-derived character even after lattice stabilization has been taken place.

A systematic variation of tetragonal crystal-field parameters shows that weaker distortions fail to reproduce the experimental line shape, while stronger distortions do not yield further improvement (Figure S7). This sensitivity substantiates that the chosen distortion magnitude captures the effective local symmetry lowering associated with the stabilized excited state. It should be noted that these parameters should be interpreted as spectroscopic descriptors of local lattice rearrangement rather than as measures of static structural distortions.

The robustness of the late-time assignment is supported by comparison with calculated spectra for distorted Fe^2+^ configurations. As shown in Figure S7c,d, even when comparable distortion magnitudes are applied, Fe^2+^-based models do not reproduce the experimental transient spectra in terms of energy positions, relative intensities, or overall multiplet structure. This exclusion decisively demonstrates that the long-lived photoexcited state does not correspond to sustained reduction of the iron center. Rather, the data support a picture in which charge localization is accompanied by lattice distortion while the formal Fe^3+^ valence is preserved.

Overall, the late-time spectra indicate the formation of a polaronic excited state, characterized by local symmetry lowering at Fe^3+^ sites and partial retention of LMCT-derived electronic character. The smooth evolution of spectral weights from the intermediate regime into the stabilized late-time response emphasizes the continuous nature of the relaxation pathway. Charge localization and lattice reorganization proceed cooperatively, culminating in a lattice-stabilized configuration that is electronically distinct from both the initial LMCT state and any fully reduced Fe^2+^ species.

The persistence of this polaronic state over several picoseconds indicates the efficiency with which the perovskite lattice accommodates photoinduced charge localization. In the context of the earlier comparison with α-Fe_2_O_3_, the present results emphasize that the corner-sharing connectivity of the FeO_6_ network in LaFeO_3_ not only prolongs the lifetime of the charge-transfer excitation but also enables rapid and effective lattice stabilization. The iron 3*p* edge, with its heightened sensitivity to local symmetry changes, thus gives access to the final stages of polaron formation that remain less apparent in higher-energy core-level probes.

## 3 Conclusion

In this work, femtosecond XUV absorption spectroscopy at the iron 3*p* edge was employed to resolve the ultrafast electronic and lattice dynamics of photoexcited LaFeO_3_ with element and symmetry sensitivity. By combining time-resolved measurements with crystal-field multiplet calculations, we identify a continuous relaxation pathway that links impulsive LMCT excitation to the formation of a lattice-stabilized polaronic state, without evidence for long-lived Fe^2+^ formation.

The early-time response is governed by the population of a mixed LMCT state, which is prevalent in t_2g_^3^e_g_^3^ (^5^ *E*) character. On subpicosecond time scales, lattice vibrations associated with local distortions around the carrier promote partial charge localization and symmetry lowering at Fe^3+^ sites. This behavior is indicative of the emergence of a polaronic state. At later delays, the transient spectra are dominated by distorted Fe^3+^ configurations with residual LMCT-derived character, demonstrating that lattice reorganization stabilizes the excited state while preserving the formal oxidation state of iron. Comparison with α-Fe_2_O_3_ reveals distinct material dependence in both charge-transfer relaxation and polaron-formation dynamics, manifesting the roles of lattice topology and iron–oxygen covalency.

Beyond elucidating the relaxation pathway in LaFeO_3_, these results are relevant for a broad class of functional oxides in which charge–lattice coupling governs performance, including photocatalysis, photoelectrochemical energy conversion, and resistive switching devices.^4,5^ More broadly, femtosecond XUV absorption spectroscopy at the 3*p* edge paves the way for probing charge localization and lattice response in correlated materials, complementing higher-energy core-level spectroscopies with enhanced sensitivity to local symmetry and covalency.^32–34^

## 4 Methods

### 4.1 XUV Experiments

Transient absorption of textured LaFeO_3_ thin films was performed at the X-FAST instrument^29^ at the University of Wisconsin–Madison, USA. The sample was excited by a 3 μJ, 70 fs pulse with a central wavelength of 400 nm (Figure 1). The spot sizes of the pump and probe beams were approximately 200 and 80 μm, respectively. The N_2_ gas flows between the membranes, introduced through a 1/16 in. tubing and a 250 μm diameter entrance hole in the gas cell. It then passes over the sample and exits through a 250 μm hole into the sample chamber. For transient absorption experiments, a flow rate of approximately 15 standard cubic centimeters per minute (SCCM) is maintained, with an estimated gas cell pressure of about 10 Torr. Thermal accumulation is mitigated by the low duty cycle and the continuous nitrogen gas flow, which provides efficient heat dissipation. No irreversible spectral changes, baseline drift, or degradation were observed over extended acquisition times. The introduction of N_2_ gas in the cell reduces XUV transmission by approximately 10%. The average photon flux detected at the charge-coupled device (CCD) between 40 and 72 eV was 1.8 × 10^5^ photon/pulse.^29^ The temporal resolution of the experiment was approximately 85 fs. To visualize the ultrafast dynamics, the data were plotted as a 2D color map using 120 contour levels in Figure 2b, providing a visually smooth representation of the underlying matrix without the need for explicit numerical smoothing. The matrix was transposed to properly align the time and energy dimensions and scaled to enhance contrast.

### 4.2 Pulsed Laser Deposition (PLD)

The textured LaFeO_3_ thin film was grown by pulsed laser deposition (PLD) at the MESA+ Institute for Nanotechnology of the University of Twente, The Netherlands. The Si_3_N_4_ membranes were purchased from Norcada with a nitride thickness of 50 nm, a window size of 1.0 × 1.0 mm^2^, a frame size of 5.0 × 5.0 mm^2^, and a frame thickness of 200 μm (Figure 2a). The Si_3_N_4_ membranes were mounted on a custom-made shadow mask sample holder. The O_2_ pressure during the growth was 0.01 mbar. The growth temperature was 700 °C, measured with a K-type thermocouple inside a resistive substrate heater. The fluence was 1.9 J cm^–2^, the target–substrate distance was 5 cm, and the frequency was 2 Hz. The thickness of the LaFeO_3_ thin film was determined to be 11 nm using the XRD results (Figure S3a–c).

### 4.3 X-ray Diffraction (XRD)

The X-ray diffraction (XRD) measurements were performed using PANalytical X’Pert Pro with a nonmonochromatic Cu anode and a Ni filter. The highly textured growth of the LaFeO_3_ thin film on the Ca_2_Nb_3_O_10_ nanosheet-templated Si_3_N_4_ membrane, resulting in local epitaxy, was confirmed by XRD results (Figure S3a–c).

### 4.4 Atomic Force Microscopy (AFM)

The topography of the Ca_2_Nb_3_O_10_ nanosheets and the grown film was characterized by atomic force microscopy (AFM) using a Veeco Dimension Icon AFM in tapping mode in the air. The oscillating cantilever is a Tespa-V2 cantilever (Bruker, Netherlands) with a silicon tip with a nominal radius of 20 nm. The AFM images were obtained using the Nanoscope software and treated using the Gwyddion software,^35^ shown in Figure S2.

### 4.5 Multiplet Calculations

The multiplet calculations were carried out using Quanty^28,36,37^ and our in-house developed CTM4XAS^9,25,38^ program. The energy-dependent broadening is applied to the 3*p* XAS spectra, emphasizing the shorter Auger lifetime at higher energies due to the increased number of decay channels.^26^ The spectrum is linearly broadening from 0.1 to 1.5 eV between 55 and 57.5 eV, while a Gaussian broadening of 0.1 eV is introduced to match the experimental resolution. To capture the intrinsic asymmetry arising from the short-lived nature of the XAS final state, an asymmetric Fano broadening of 1.6 eV is applied to the main peak. This broadening indicates the coupling of the discrete excited state to the continuum, as the final state decays predominantly through Auger processes.^26^ The 3*p* core level decays more than 99% via Auger.^23^ Due to the shared initial and final states, the overlap of these transitions gives rise to interference effects, resulting in an asymmetric line shape in the spectrum.

## Supplementary Material



## Data Availability

All data are available from the corresponding authors upon reasonable request.

## Notes

Additional experimental details on the preparation of Ca_2_Nb_3_O_10_ nanosheet templates using the Langmuir–Blodgett technique, AFM and XRD characterization, background subtraction procedures, complementary multiplet calculations, and kinetic trace fitting (PDF)
