# Supplementary material for: Deciphering Femtosecond Charge–Lattice Dynamics in Textured LaFeO3 Thin Films
Source: ACS Nano. 2026 Aug 26;20(35):24366–75. doi: 10.1021/acsnano.6c04204 (PMC13565294; doi:10.1021/acsnano.6c04204)
Supplement: Supplementary file 1 [file nn6c04204_si_001.pdf]

Supporting Information

Deciphering femtosecond Charge–Lattice dynamics in textured $LaFeO_3$ thin films

Masoud Lazemi[1,2,3,*], Fabian J. Mohammad[1], Ryan Ash[4], Zain Abhari[4], Roberta Candela[4], Hans J.F.A. Blankesteijn[1], Emma van der Minne[2], Yorick A. Birkhölzer[2], Miguel Segovia Guzmán[3], Moritz Nunnenkamp[2], Phu Tran Phong Le[2], Sang Han Park[5], Abhishek Katoch[6], Soonnam Kwon[5], Christoph Baeumer[2], Gertjan Koster[2], Arnaud Rouzée[3], Uwe Bergmann[4*], and Frank M. F. de Groot[1*]

[1]Materials Chemistry and Catalysis, Debye Institute for Nanomaterials Science, Utrecht University, Universiteitsweg 99, 3584 CG Utrecht, The Netherlands

[2]MESA+ Institute for Nanotechnology, University of Twente, P. O. Box 217, Enschede, 7500 AE, The Netherlands

[3]Max Born Institute for Nonlinear Optics and Short Pulse Spectroscopy, Max-Born-Straße 2A, 12489 Berlin, Germany

[4]Department of Physics, University of Wisconsin–Madison, Madison, Wisconsin, 53706-1390, USA

[5]Pohang Accelerator Laboratory, Pohang, Gyeongbuk 37673, Republic of Korea

[6]Department of Chemistry, Yonsei University, Seoul 03722, Republic of Korea

Corresponding authors: m.lazemi@uu.nl, ubergmann@wisc.edu, and F.M.F.deGroot@uu.nl*

1. Monolayer deposition of $Ca_2Nb_3O_{10}$ nanosheets using the Langmuir–Blodgett technique

As shown in **Fig. S1**, the Si_3N_4 membrane was first mounted on a quartz glass support and treated with oxygen plasma to render the surface hydrophilic. This modification promotes uniform nanosheet coverage during the deposition process. The Langmuir–Blodgett (LB) bath was then filled with a tetrabutylammonium hydroxide (TBAOH) aqueous solution, into which the quartz-supported Si_3N_4 membrane was carefully immersed. $Ca_2Nb_3O_{10}$ nanosheets were synthesized following the procedure outlined in our previous report.[1] The synthesized nanosheets were introduced into the TBAOH solution using a micropipette. To ensure homogeneous dispersion and minimize aggregation, the solution was allowed to rest for 10 minutes prior to initiating the LB deposition process.

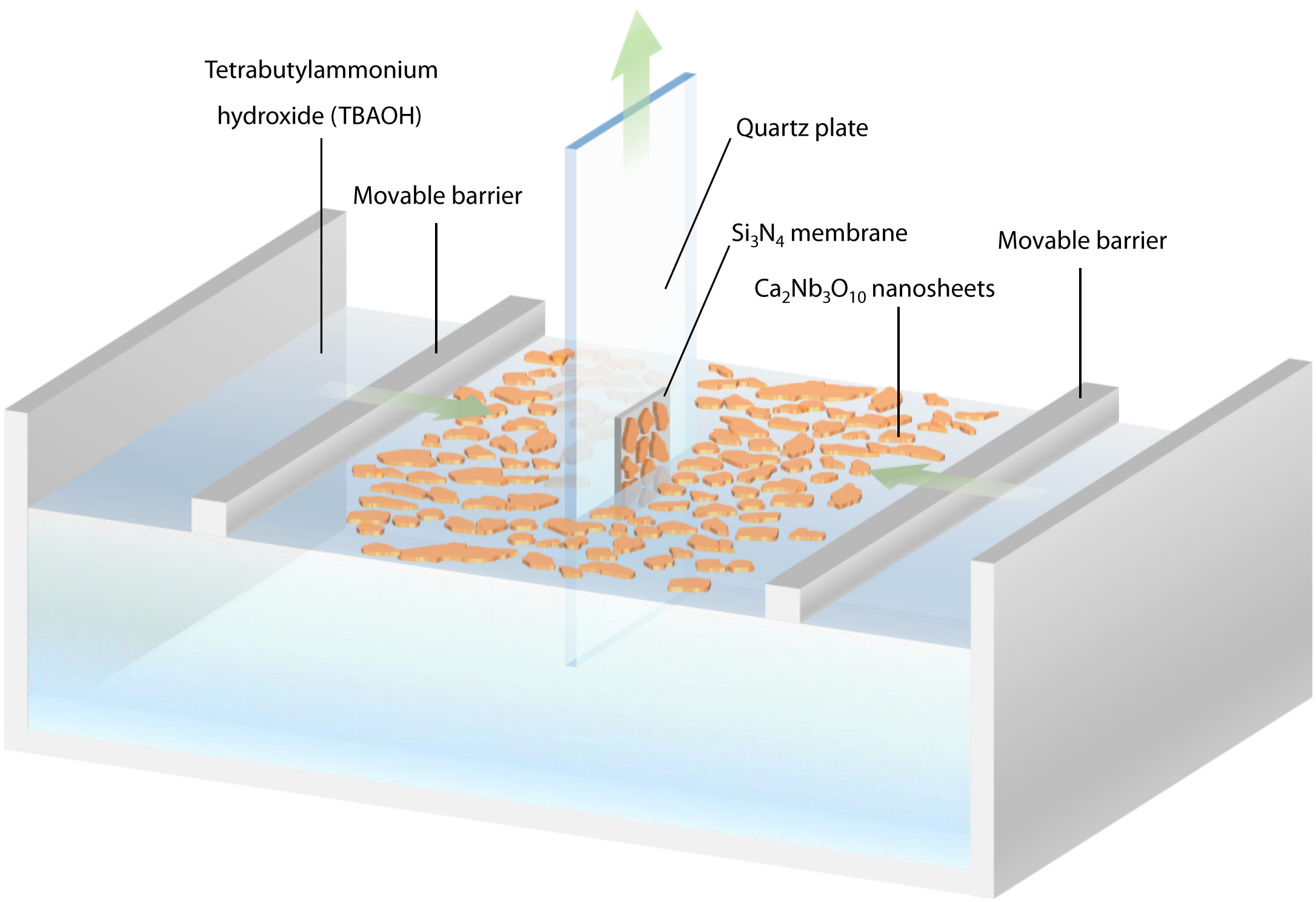

Fig. S1. Langmuir–Blodgett deposition of $Ca_2Nb_3O_{10}$ nanosheets. Schematic illustration of the Langmuir–Blodgett deposition process used to form a monolayer of $Ca_2Nb_3O_{10}$ nanosheets on a Si_3N_4 membrane. The nanosheets are first spread at the air–TBAOH interface and subsequently compressed to form a closely packed monolayer, which is then transferred onto the Si_3N_4 membrane by vertical dipping. This procedure yields a uniform nanosheet template that serves as a crystalline seed layer for the oriented growth of $LaFeO_3$ thin films.

2. Atomic force microscopy (AFM)

Fig. S2a shows an AFM image of the Si_3N_4 membrane surface, which is uniformly covered with $Ca_2Nb_3O_{10}$ nanosheets. These nanosheets form a dense and continuous layer, ensuring conformal coverage over the amorphous membrane. They exhibit lateral widths ranging from 1 to 5 µm and a thickness of approximately 1–2 nm, consistent with monolayer exfoliation and deposition. This nanosheet layer serves as a crystalline template, facilitating the subsequent epitaxial growth of $LaFeO_3$. **Fig. S2b** presents the AFM image after $LaFeO_3$ deposition, showing a textured film morphology indicative of oriented film growth on the nanosheet substrate. All AFM measurements were performed at the edge of the Si_3N_4 window to avoid puncturing or damaging the delicate membrane area while still capturing representative surface features.

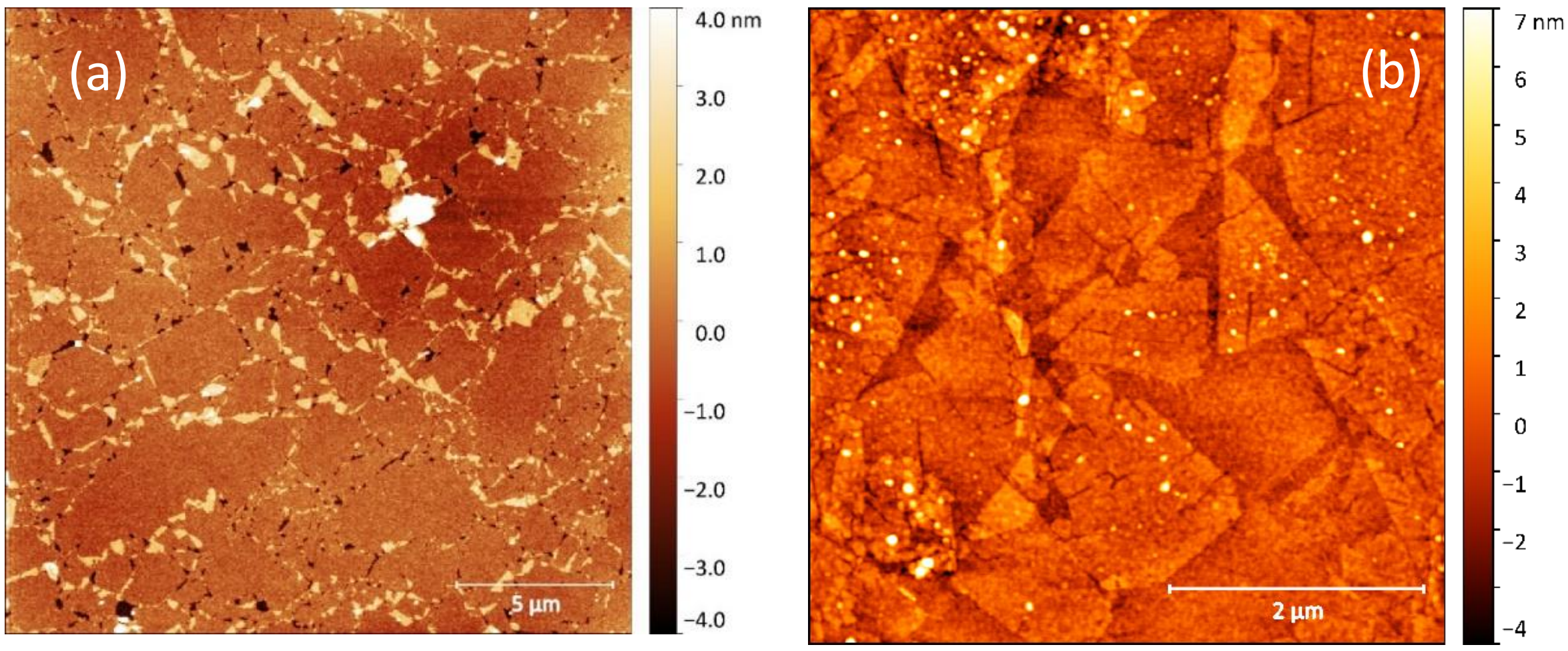

Fig. S2. Nanosheet templating enables textured oxide growth on membranes. Atomic force microscopy confirms nanosheet templating and film continuity on Si_3N_4 membranes. The AFM image of the **(a)** as-deposited $Ca_2Nb_3O_{10}$ nanosheets on a Si_3N_4 membrane **(b)** after $LaFeO_3$ deposition.

3. X-ray diffraction (XRD)

Fig. S3a presents the X-ray diffraction (XRD) pattern of $LaFeO_3$ grown on a $Ca_2Nb_3O_{10}$-nanosheet-templated Si_3N_4 membrane. The measurements were performed using a PANalytical X'Pert Pro diffractometer equipped with a non-monochromated Cu anode source and a Ni filter positioned in front of a PIXcel detector. **Figs. S3b** and **S3c** show a high-resolution scan around the (002) reflection and the corresponding rocking curve, respectively. The full width at half maximum (FWHM) of the rocking curve was measured to be 2.2°, indicating a favorable degree of out-of-plane alignment and suggesting that the nanosheet template promotes preferential orientation during film growth. The film thickness was estimated to be approximately 11 nm based on kinetic diffraction simulations, following the methodology described elsewhere.[2]

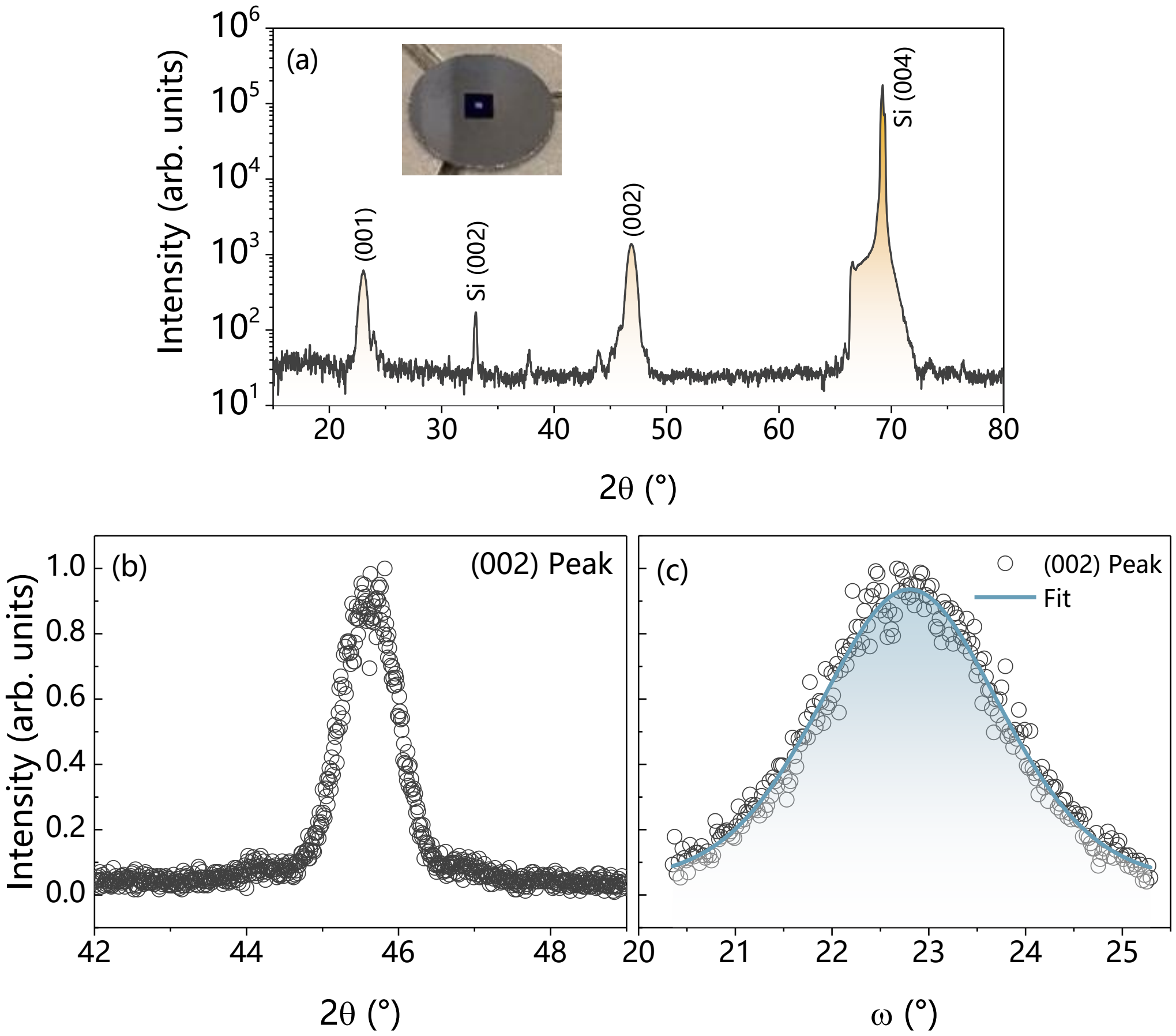

Fig. S3. Structural characterization of textured $LaFeO_3$ films. X-ray diffraction confirms oriented growth of $LaFeO_3$ on nanosheet-templated membranes. **(a)** XRD of $LaFeO_3$ on $Ca_2Nb_3O_{10}$-nanosheet-templated Si_3N_4 membrane. The inset shows the sample on a custom-made stainless steel shadow mask holder. The size of the sample is 5×5 mm^2. The membrane window is 1×1 mm^2. **(b)** Normalized high-resolution XRD at (002) peak and **(c)** corresponding rocking curve.

4. Background subtraction

Fig. S4 plots the ground state XAS of pristine and $Ca_2Nb_3O_{10}$-templated Si_3N_4 membrane. The latter was subtracted from the ground state XAS spectrum shown in the top panel of **Fig. 2c**. In transmission spectroscopy, when both the sample and reference spectra are available—specifically, the film deposited on the nanosheet substrate and the bare nanosheet substrate—the most effective background correction method is division rather than subtraction. Dividing the film-plus-substrate spectrum by the substrate-only spectrum compensates for transmission losses introduced by the substrate alone, thereby isolating the contribution from the film. This normalization step ensures that the resulting spectrum reflects only the optical response of the film itself.

In contrast, simple subtraction fails to account for the multiplicative nature of transmission, as it assumes an additive relationship. This can result in negative values or spectral distortions, particularly when the substrate exhibits strong absorption features. To avoid these issues, we apply a division-based correction (Eq. S1), as described above. The corrected spectrum is presented in the top panel of **Fig. 2c**.

$$XAS_{film\ only} = \frac{XAS_{film+nanosheet+substrate}}{XAS_{nanosheet+substrate}} \qquad \text{(Eq. S1)}$$

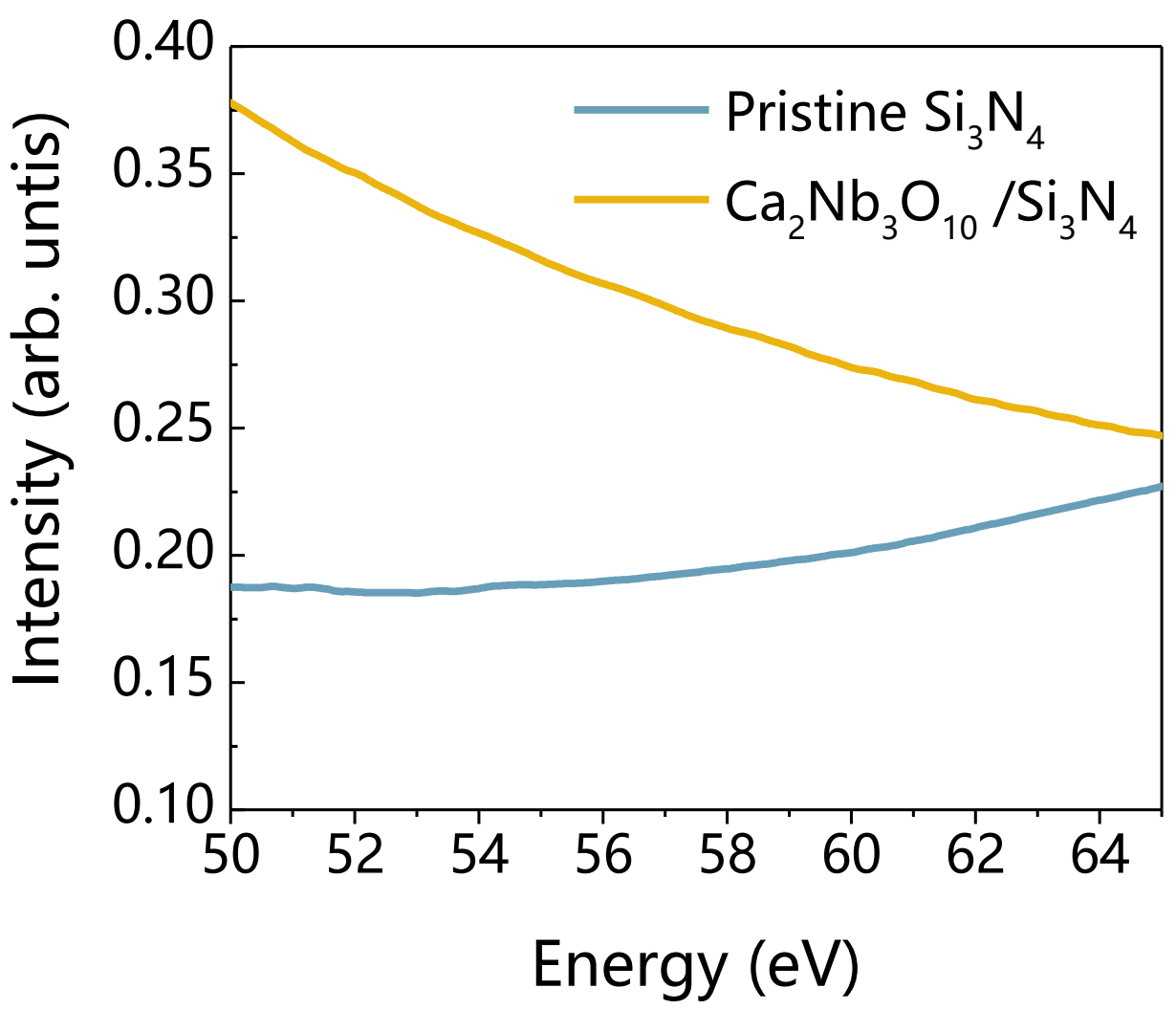

Fig. S4 Ground state XAS of pristine and $Ca_2Nb_3O_{10}$-templated Si_3N_4 membrane.

5. Ligand-to-metal charge transfer (LMCT)

Fig. S5 shows the calculated XAS of $t_{2g}^3 e_g^3$ (^5E) and $t_{2g}^4 e_g^2$ ($^5T_2$) states using CTM4XAS[3–5] and Quanty.[6–8] The transient spectra are depicted in **Fig. 3(a)**. The LMCT calculation parameters are reported in **Table S1**.

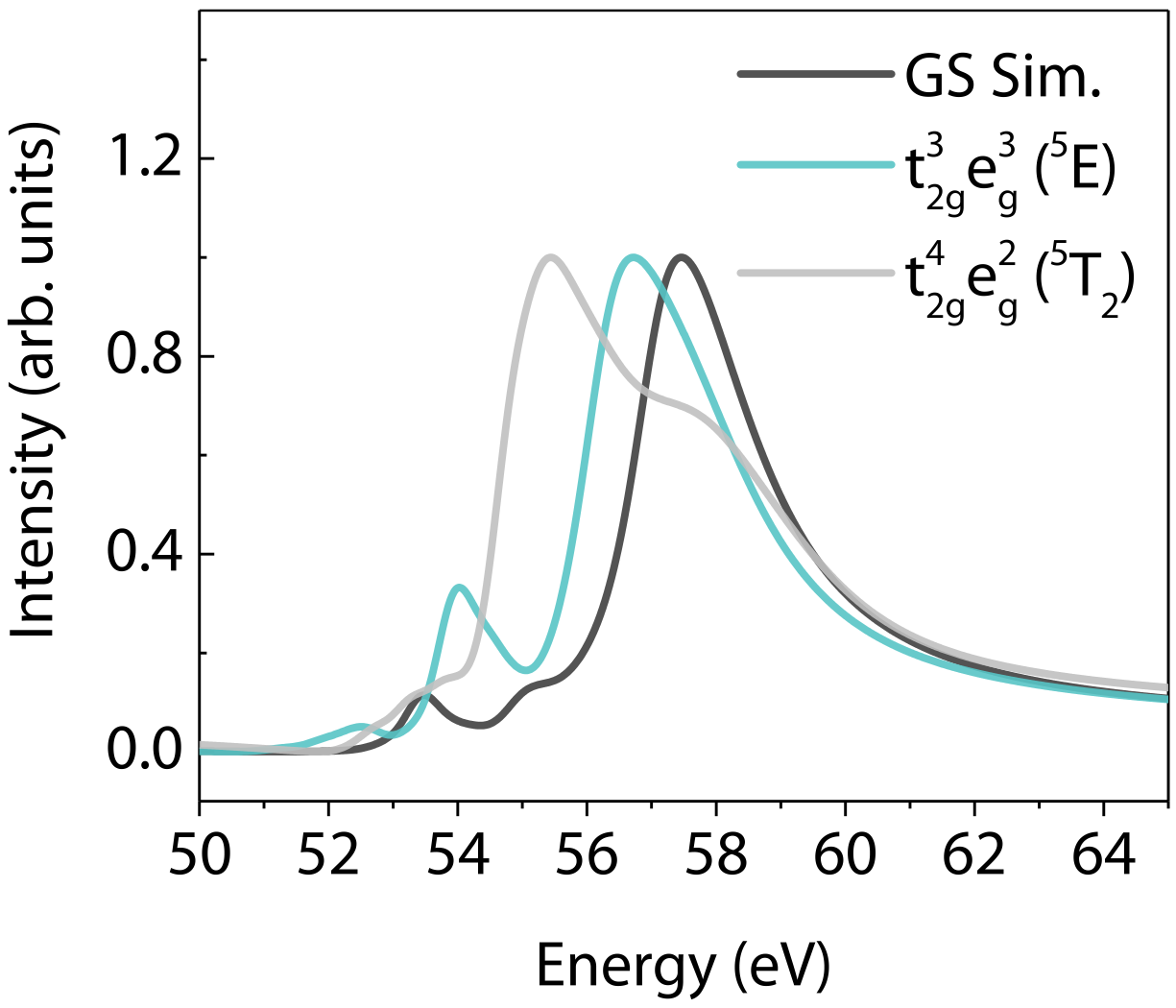

Fig. S5. Multiplet spectra for LMCT configurations. Calculated iron 3*p* XAS spectra establish reference multiplet fingerprints for LMCT configurations. Calculated XAS of $t_{2g}^4 e_g^2$ ($^5T_2$) and $t_{2g}^3 e_g^3$ (^5E) states along with the ground state (GS).

Table S1. The parameters used in the calculations, Slater integrals, and spin-orbit couplings. A crystal-field splitting parameter of 10Dq = 1.8 eV was employed, and the Slater integrals were scaled to 80% of their Hartree–Fock values.

			F_{dd}^2	F_{dd}^4	ζ_{2p}	F_{pd}^2	G_{pd}^1	G_{pd}^3
Fe^{3+} ($3d^5$)	Initial state	$2p^63d^5$	12.043	7.535				
	Final state	$2p^53d^6$	12.091	7.565	0.969	13.050	16.176	9.854
Fe^{2+} ($3d^6$)	Initial state	$2p^63d^6$	10.965	6.815				
	Final state	$2p^53d^7$	11.069	6.882	0.943	12.316	15.311	9.253
Fe^{4+} ($3d^4$)	Initial state	$2p^63d^4$	13.030	8.198				
	Final state	$2p^53d^5$	13.039	8.202	1.003	13.717	16.912	10.388

6. Low- and intermediate-spin iron states

As illustrated in **Fig. S6a,b**, we explored the possibility of low- and intermediate-spin iron states by calculating their XAS spectra. The low-spin state requires extreme Fe–O bond contraction, while the intermediate-spin state requires strong structural distortions. However, these transitions do not occur significantly under our experimental conditions. Instead, our results are more consistent with LMCT, where Fe^{3+} is transiently reduced to Fe^{2+}, without strong Fe–O contraction or Jahn-Teller distortions.

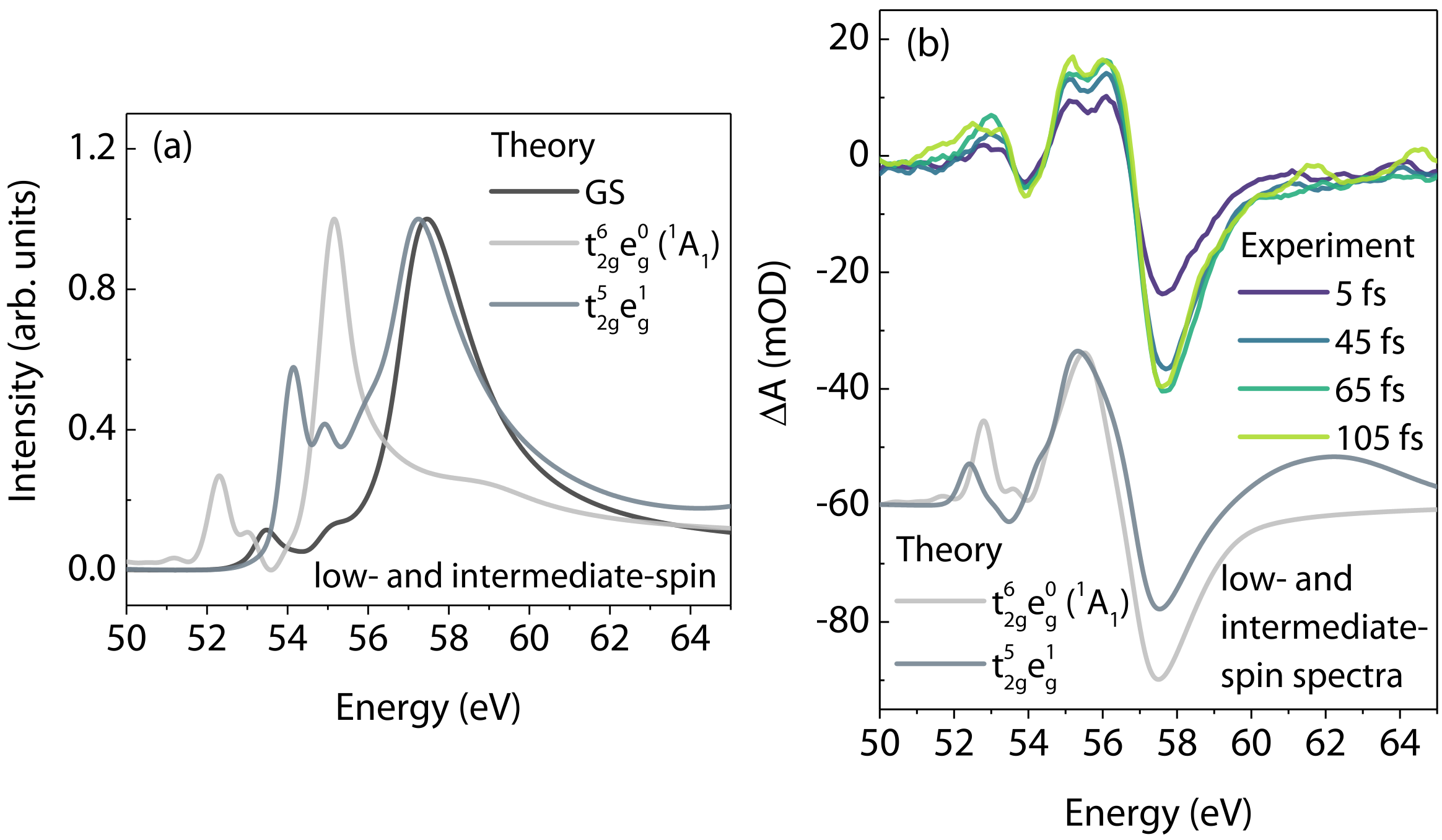

Fig. S6. Exclusion of spin-state transitions. Calculated low- and intermediate-spin iron configurations are inconsistent with the experimental transient spectra. Calculated low- and intermediate-spin iron states, $t_{2g}^6 e_g^0$ $(^1A_1)$ and $t_{2g}^5 e_g^1$ **(a)** XAS spectra **(b)** transient spectra.

7. Fe^{2+} and Fe^{3+} distortions

Fig. S7a,b plot the calculated XAS and transient spectra considering Fe^{2+} and Fe^{3+} distortions. The Fe^{2+} distortions were calculated considering D_s = 0.1 eV, $D_s = D_t$ = 0.2 eV, and D_t = 0.1 eV, while the Fe^{3+} distortions were calculated with D_s = 0.1 eV, $D_s = D_t$ = 0.1 eV, and $D_s = D_t$ = 0.2 eV. The corresponding transient spectra of Fe^{3+} distortions are shown in **Fig. 4**.

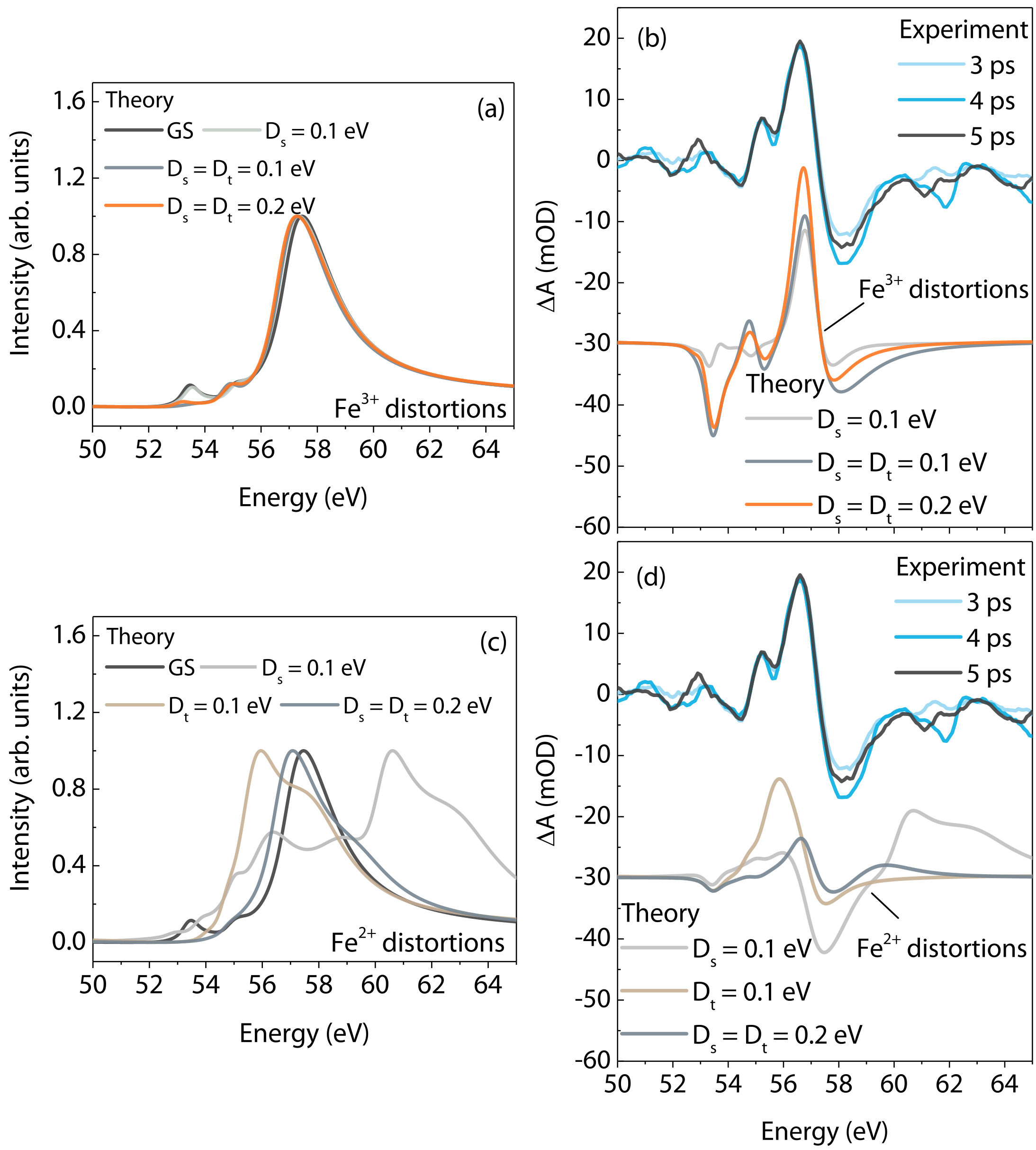

Fig. S7. Sensitivity of iron 3*p* spectra to lattice distortion. Crystal-field multiplet calculations quantify the spectral signatures of Fe^{3+} and Fe^{2+} lattice distortions. Calculated XAS spectra considering **(a)** GS and Fe^{3+} distortions. The Fe^{3+} distortions were calculated considering D_s = 0.1 eV, $D_s = D_t$ = 0.1 eV, and $D_s = D_t$ = 0.2 eV (10Dq = 1.8 eV). The corresponding transient spectra are shown in Fig. 4. **(b)** Transient spectra at the delay times of 3, 4, and 5 ps, along with the calculated spectra considering Fe^{3+} distortions. The Fe^{3+} distortions were calculated considering D_s = 0.1 eV, $D_s = D_t$ = 0.1 eV, and $D_s = D_t$ = 0.2 eV. **(c)** GS and Fe^{2+} distortions and **(d)** Transient spectra at the delay times of 3, 4, and 5 ps, along with the calculated spectra considering Fe^{2+} distortions. The Fe^{2+} distortions were calculated considering D_s = 0.1 eV, D_t = 0.1 eV, and $D_s = D_t$ = 0.2 eV.

8. *d-d* excitations

Fig. S8a,b show the calculated XAS and transient spectra considering the *d-d* excitations, corresponding to intra-atomic electron transitions between iron 3*d* orbitals, for instance, $t_{2g} \rightarrow e_g$ within the FeO_6 crystal field. However, *d-d* transitions are usually dipole-forbidden in centrosymmetric Fe^{3+} coordination, making them optically weak. Moreover, the crystal field splitting (Δ) in $LaFeO_3$ is typically less than 3 eV, meaning that the 400 nm (3.1 eV) pump is likely too energetic for a simple $t_{2g} \rightarrow e_g$ transition. The *d-d* excitations are more common in visible or near-infrared absorption rather than UV or blue wavelengths.[9]

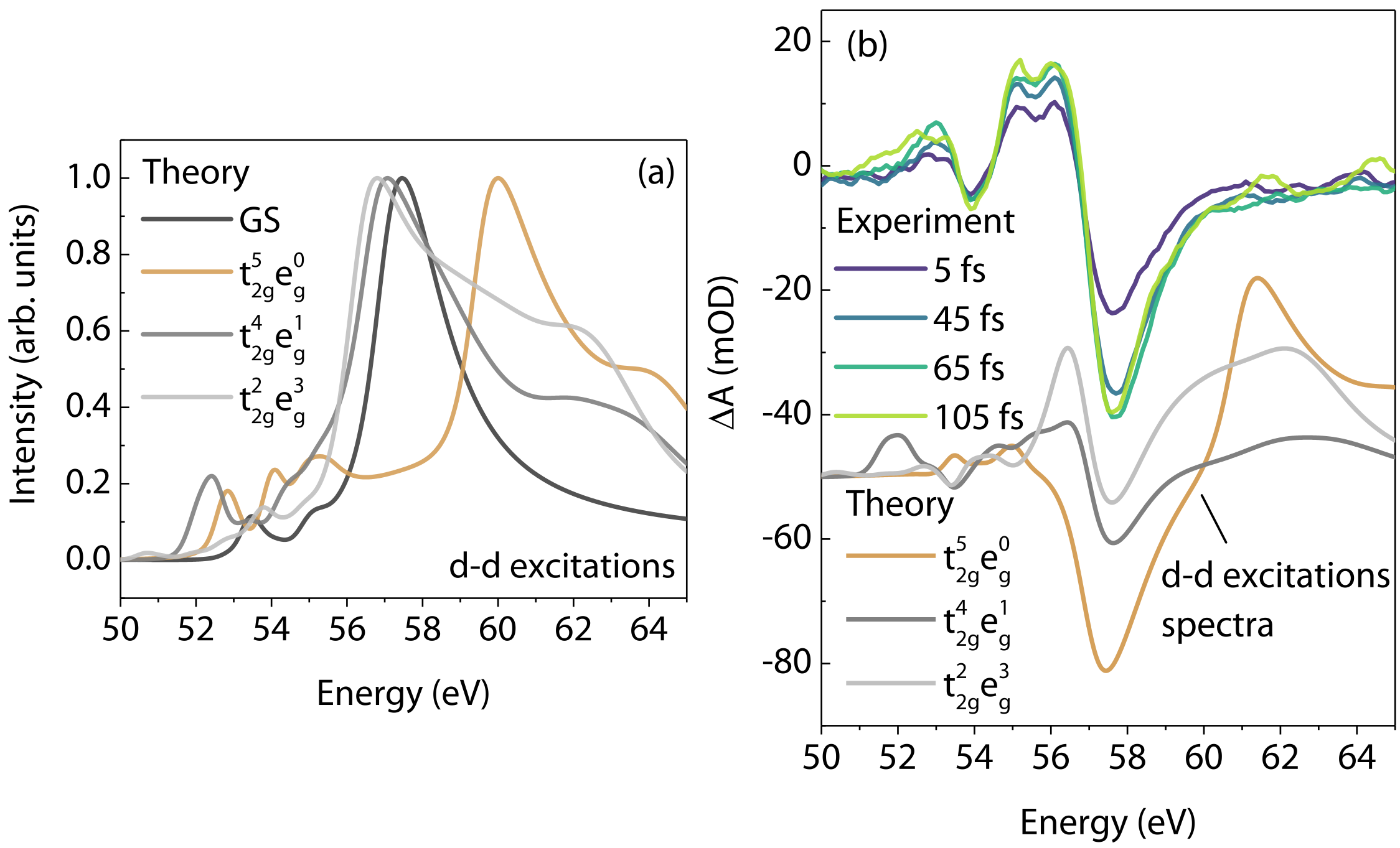

Fig. S8. Exclusion of alternative excitation pathways. Calculated d–d excitation spectra do not reproduce the experimental transient response. Calculated **(a)** XAS and **(b)** transient spectra considering the *d-d* excitations of $t_{2g}^5 e_g^0$, $t_{2g}^4 e_g^1$, and $t_{2g}^2 e_g^3$.

9. Metal-to-metal charge transfer (MMCT)

Fig. S9a,b illustrate the calculated MMCT spectra, referring to electron transfer between iron sites, such as:

$Fe^{3+} + Fe^{3+} \rightarrow Fe^{2+} + Fe^{4+}$

In $LaFeO_3$, all iron atoms are nominally Fe^{3+}, hence, there is no intrinsic Fe^{2+}/Fe^{4+} mixed valency to facilitate MMCT. On the other hand, the Fe^{3+}–Fe^{3+} *d-d* exchange interaction is relatively weak, meaning that direct charge transfer between iron sites requires a larger excitation energy. Moreover, the iron sites in perovskite $LaFeO_3$ are separated by oxygen anions, further suppressing direct MMCT.

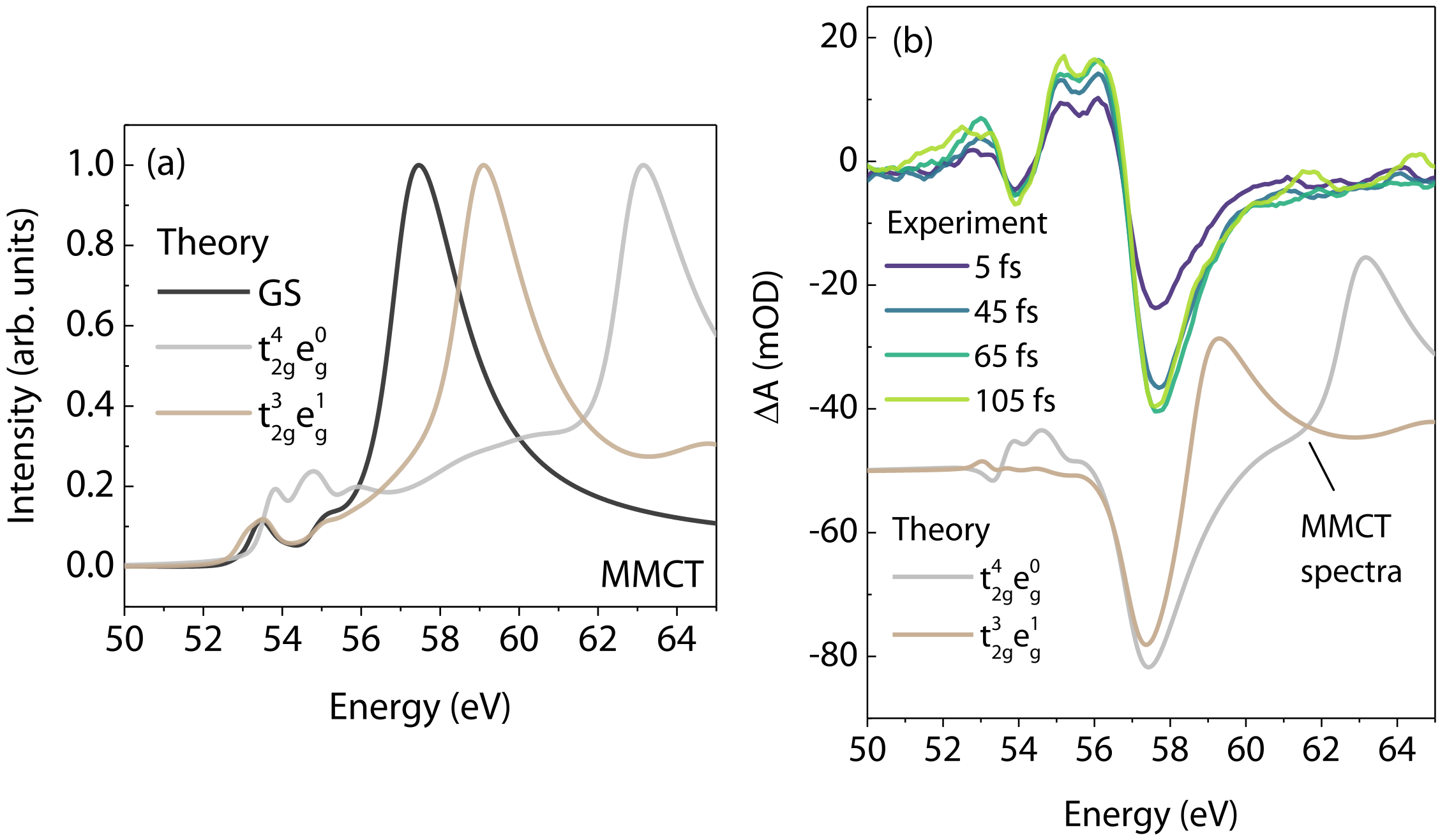

Fig. S9. Exclusion of alternative excitation pathways. Metal-to-metal charge-transfer (MMCT) configurations are inconsistent with the observed spectra. Calculated **(a)** XAS and **(b)** transient spectra considering the MMCT states of $t_{2g}^3 e_g^1$ and $t_{2g}^4 e_g^0$.

10. Fitting kinetic traces

The following equation was used to fit the kinetic traces,[10–13] which describes a multi-exponential decay response convolved with a Gaussian instrument response function (IRF).

$$I(\Delta t) = \frac{1}{2}\left(1 - erf\left(\frac{-(\Delta t - t_c)}{\sqrt{2}\frac{\tau_{ref}}{2.355}}\right)\right)\left(I_1 exp\left(-\frac{\Delta t - t_c}{\tau_1}\right) + I_2 exp\left(-\frac{\Delta t - t_c}{\tau_2}\right) + I_3 exp\left(-\frac{\Delta t - t_c}{\tau_3}\right)\right) + I_{offset}$$

(Eq. S2)

Δt is the time delay between the pump and probe pulses. t_c is the time-zero or temporal center of the response. τ_{ref} is the FWHM of the instrument response function (IRF), related to the time resolution. *erf* is the error function, which arises when convolving a step or exponential function with a Gaussian. I_1, I_2, and I_3 are the amplitudes (or contributions) of three different exponential decay processes. τ_1, τ_2, and τ_3 are the corresponding decay lifetimes. I_{offset} is a constant offset parameter accounting for static background contributions in the transient signal, such as residual baseline offsets or long-lived components beyond the experimental time window. It ensures that the fitted exponential terms describe only the dynamic photoinduced response.

10.1. Feature A (55 eV)

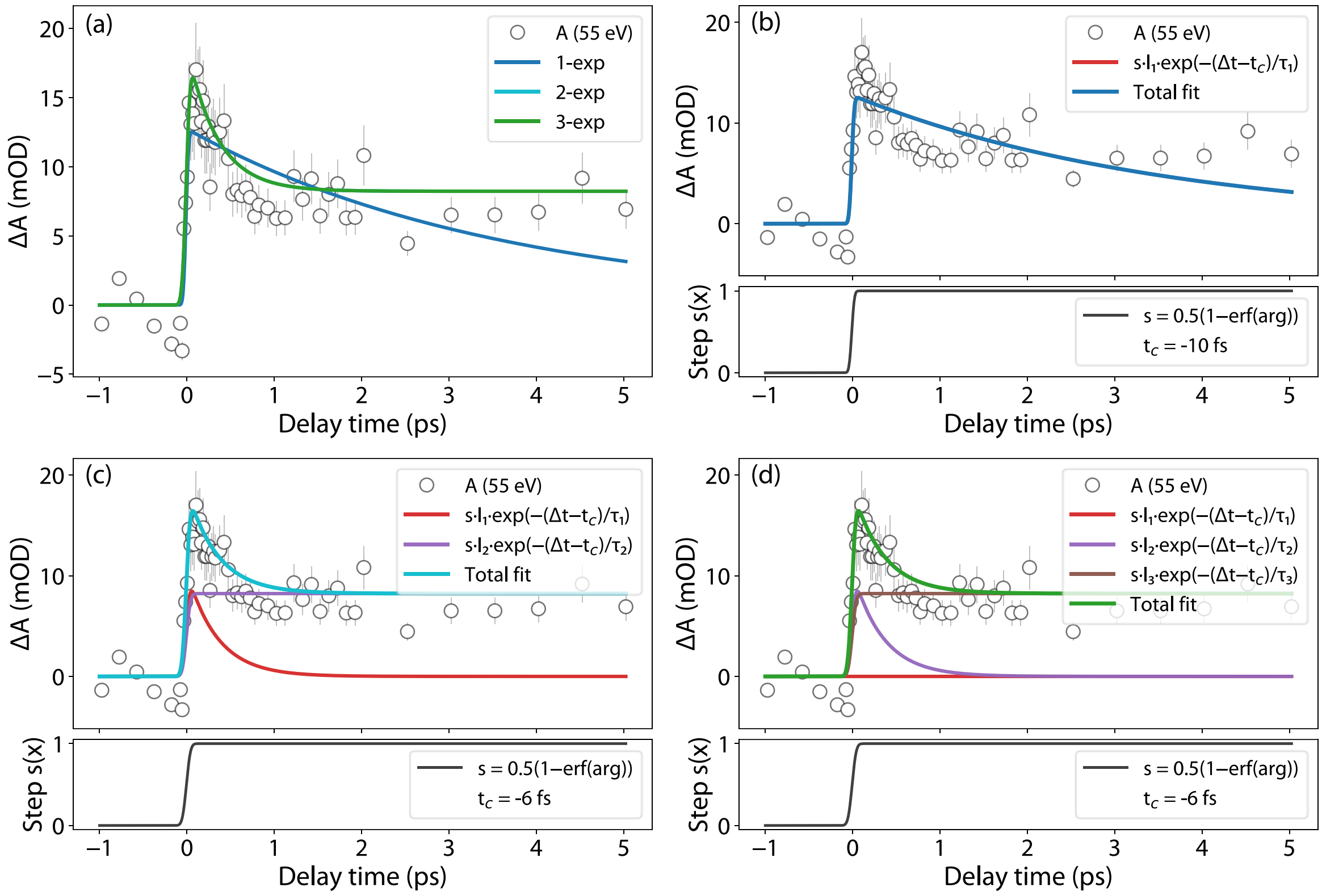

Fig. S10. Kinetic analysis. Kinetic trace at feature A (55 eV) using 400 nm excitation **(a)** Comparison between single-, double-, and triple-exponential fit. **(b)** single-exponential **(c)** double-exponential **(d)** triple-exponential. The vertical lines show the error bars. In panel (a), the resulting double- and triple-exponential fits overlap.

Table S2. Summary of fitting parameters extracted from kinetic traces at feature A (55 eV)

Model	I_1	I_2	I_3	I_{offset}	τ_1	τ_2	τ_3	τ_c (fs)	τ_{ref} (fs)
1-exp	0.013	-	-	≈0	3.60 ps	-	-	-10	55
2-exp	0.011	0.008	-	-0.001	349 fs	6.52 μs	-	-6	86
3-exp	≈0	0.011	0.008	-0.001	-	349 fs	5.03 μs	-6	86

10.2. Feature B (56.5 eV)

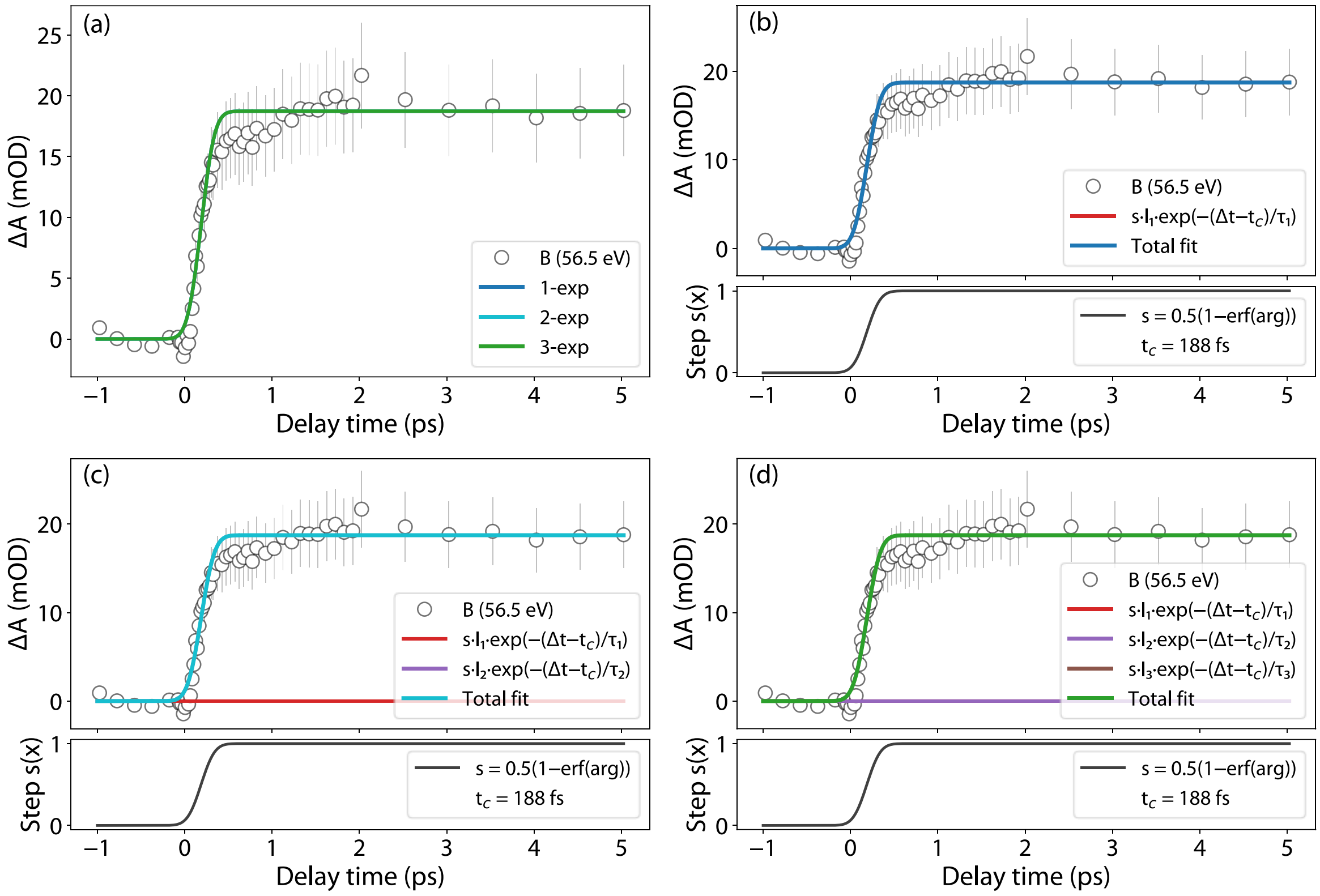

Fig. S11. Kinetic analysis. Kinetic trace at feature B (56.5 eV) using 400 nm excitation **(a)** Comparison between single-, double-, and triple-exponential fit. **(b)** single-exponential **(c)** double-exponential **(d)** triple-exponential. In panel (a), the resulting fits are overlapping.

Table S3. Summary of fitting parameters extracted from kinetic traces at feature B (56.5 eV)

Model	I_1	I_2	I_3	I_{offset}	τ_1	τ_2	τ_3	τ_c (fs)	τ_{ref} (fs)
1-exp	0.019	-	-	-0.0007	6.18 µs	-	-	188	288
2-exp	≈0	0.019	-	-0.0007	-	17.3 µs	-	188	288
3-exp	≈0	≈0	0.019	-0.0007	-	-	8.53 µs	188	288

10.3. Feature C (57.5 eV)

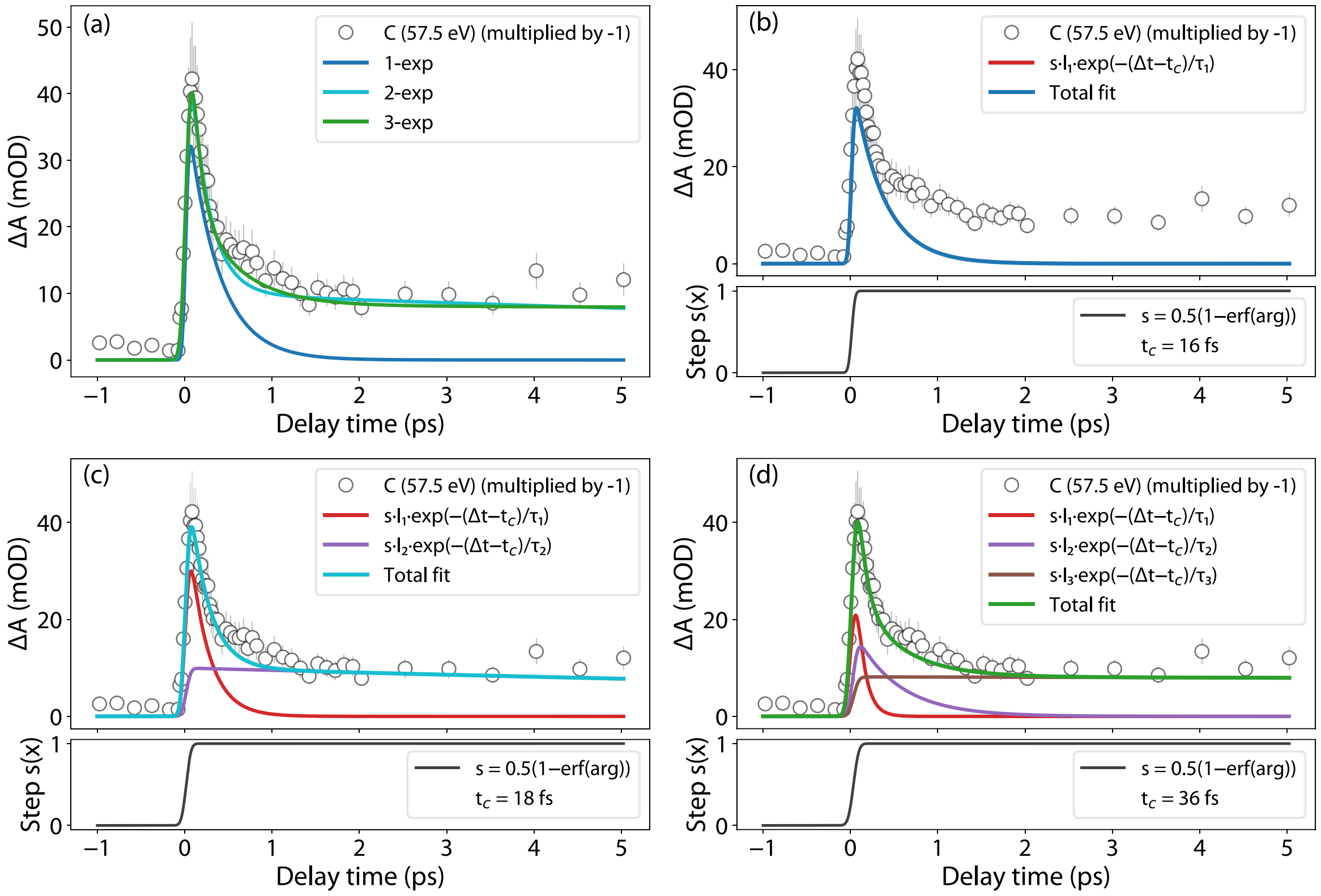

Fig. S12. Kinetic analysis. Kinetic trace at feature C (57.5 eV) using 400 nm excitation **(a)** Comparison between single-, double-, and triple-exponential fit. **(b)** single-exponential **(c)** double-exponential **(d)** triple-exponential. The data has been multiplied by -1 for fitting purposes.

Table S4. Summary of fitting parameters extracted from kinetic traces at feature C (57.5 eV)

Model	I_1	I_2	I_3	I_{offset}	τ_1	τ_2	τ_3	τ_c (fs)	τ_{ref} (fs)
1-exp	0.039			0.008	347 fs	-	-	16	71
2-exp	0.043	0.010		0.002	218 fs	19.94 ps	-	18	100
3-exp	0.039	0.018	0.008	0.002	95 fs	502 fs	203.66 ps	36	113

11. Estimation of the number of absorbed photons per iron atom

We can follow the steps below to estimate the number of absorbed photons per iron atom in $LaFeO_3$, considering a pump power of 3 µJ, a spot size of 200 µm FWHM, and an optical density of 0.5 at 400 nm.

11.1. Photon energy

The energy of a single photon with a wavelength of λ = 400 nm is:

$$E_{photon} = \frac{hc}{\lambda} \approx 4.97 \times 10^{-19} J,$$

where $h = 6.626 \times 10^{-34} J.s$ (Planck's constant) and $c = 3 \times 10^{8} m/s$ (speed of light).

11.2. Number of photons in the pulse

Given a pump energy of 3 µJ:

$$N_{Photon} = \frac{Pump\ power}{E_{photon}} = \frac{3 \times 10^{-6} J}{4.97 \times 10^{-19} J/photon} \approx 6.03 \times 10^{12}\ photons$$

11.3. Absorbed photons calculation

We assume that the optical density (OD) at 400 nm is approximately 0.5, then only 31.6 % of photons are absorbed:[14]

Absorbed Fraction = $1 - 10^{-OD} = 1 - 10^{-0.5} \approx 1 - 0.316 = 0.684$

$$N_{absorbed\ photons} = N_{Photon} \times \text{Absorbed Fraction} = (6.03 \times 10^{12}) \times (0.684)$$

$$N_{absorbed\ photons} = 4.12 \times 10^{12}$$

11.4. Fe atom calculation in the illuminated volume

The laser spot size is 200 ± 10 µm FWHM. Assuming a circular spot with a radius of 100 µm (average of 200 µm diameter), the spot area is:

$$Spot\ Area = \pi r^2 = \pi \times (100 \times 10^{-6} m)^2 \approx 3.14 \times 10^{-8} m^2$$

11.5. Calculation of the illuminated volume

Now, we can calculate the illuminated volume, considering a thickness of 11 nm resulting from the XRD measurements:

Volume = Spot Area × Thickness = $(3.14 \times 10^{-8} m^2) \times (11 \times 10^{-9} m) \approx 3.46 \times 10^{-16} m^3$

11.6. Mass of $LaFeO_3$ in the volume

The mass of $LaFeO_3$ in this volume can be calculated, given the density of $LaFeO_3$ = 6.51×10^3 kg/m^3.[15]

Mass = Density × Volume = $(6.51 \times 10^3 kg/m^3) \times (3.46 \times 10^{-16} m^3) \approx 2.25 \times 10^{-12} kg$

11.7. Number of Fe atoms

The molar mass of $LaFeO_3$ is 242.75×10^{-3} kg/mol.

Moles of $LaFeO_3$ = $\frac{Mass}{Molar\ Mass} = \frac{2.25 \times 10^{-12} kg}{242.75 \times 10^{-3} kg/mol} = 9.26 \times 10^{-12} mol$

Hence, using Avogadro's number of 6.022×10^{23} mol^{-1}:

Number of iron atoms = $(9.26 \times 10^{-12} mol) \times (6.022 \times 10^{23} mol^{-1}) = 5.58 \times 10^{12}$ iron atoms

11.8. Photon-to-atom ratio

$$\frac{Absorbed\ photons}{Number\ of\ Fe\ atoms} = \frac{4.12 \times 10^{12}}{5.58 \times 10^{12}} \approx 0.74$$

Therefore, for $LaFeO_3$ with a 200 µm FWHM spot size, 3 µJ pump energy at 400 nm, and a thickness of 11 nm, approximately 1 photon is absorbed per iron atom.

References:

(1) Le, P. T. P.; ten Elshof, J. E.; Koster, G. Shape Control of $Ca_2Nb_3O_{10}$ Nanosheets: Paving the Way for Monolithic Integration of Functional Oxides with CMOS. *ACS Appl. Nano Mater.* **2020**, *3* (9), 9487–9493. https://doi.org/10.1021/acsanm.0c02137.

(2) Lichtensteiger, C. *InteractiveXRDFit* : A New Tool to Simulate and Fit X-Ray Diffractograms of Oxide Thin Films and Heterostructures. *J Appl Crystallogr* **2018**, *51* (6), 1745–1751. https://doi.org/10.1107/S1600576718012840.

(3) Stavitski, E.; de Groot, F. M. F. The CTM4XAS Program for EELS and XAS Spectral Shape Analysis of Transition Metal L Edges. *Micron* **2010**, *41* (7), 687–694. https://doi.org/10.1016/j.micron.2010.06.005.

(4) de Groot, F. M. F.; Elnaggar, H.; Frati, F.; Wang, R.; Delgado-Jaime, M. U.; van Veenendaal, M.; Fernandez-Rodriguez, J.; Haverkort, M. W.; Green, R. J.; van der Laan, G.; Kvashnin, Y.; Hariki, A.; Ikeno, H.; Ramanantoanina, H.; Daul, C.; Delley, B.; Odelius, M.; Lundberg, M.; Kuhn, O.; Bokarev, S. I.; Shirley, E.; Vinson, J.; Gilmore, K.; Stener, M.; Fronzoni, G.; Decleva, P.; Kruger, P.; Retegan, M.; Joly, Y.; Vorwerk, C.; Draxl, C.; Rehr, J.; Tanaka, A. 2p X-Ray Absorption Spectroscopy of 3d Transition Metal Systems. *Journal of Electron Spectroscopy and Related Phenomena* **2021**, *249*, 147061. https://doi.org/10.1016/j.elspec.2021.147061.

(5) de Groot, F. Multiplet Effects in X-Ray Spectroscopy. *Coordination Chemistry Reviews* **2005**, *249* (1–2), 31–63. https://doi.org/10.1016/j.ccr.2004.03.018.

(6) Lu, Y.; Höppner, M.; Gunnarsson, O.; Haverkort, M. W. Efficient Real-Frequency Solver for Dynamical Mean-Field Theory. *Phys. Rev. B* **2014**, *90* (8), 085102. https://doi.org/10.1103/PhysRevB.90.085102.

(7) Haverkort, M. W.; Sangiovanni, G.; Hansmann, P.; Toschi, A.; Lu, Y.; Macke, S. Bands, Resonances, Edge Singularities and Excitons in Core Level Spectroscopy Investigated within the Dynamical Mean-Field Theory. *EPL* **2014**, *108* (5), 57004. https://doi.org/10.1209/0295-5075/108/57004.

(8) Haverkort, M. W.; Zwierzycki, M.; Andersen, O. K. Multiplet Ligand-Field Theory Using Wannier Orbitals. *Phys. Rev. B* **2012**, *85* (16), 165113. https://doi.org/10.1103/PhysRevB.85.165113.

(9) Hunault, M. O. J. Y.; Harada, Y.; Miyawaki, J.; Wang, J.; Meijerink, A.; de Groot, F. M. F.; van Schooneveld, M. M. Direct Observation of Cr^{3+} 3d States in Ruby: Toward Experimental Mechanistic Evidence of Metal Chemistry. *J. Phys. Chem. A* **2018**, *122* (18), 4399–4413. https://doi.org/10.1021/acs.jpca.8b00984.

(10) Lazemi, M.; Mohammad, F. J.; Park, S. H.; Katoch, A.; Blankesteijn, H. J. F. A.; Botello-Méndez, A. R.; van der Minne, E.; Birkhölzer, Y. A.; van den Bosch, I. C. G.; Kiens, E. M.; Baeumer, C.; Koster, G.; Kwon, S.; Bergmann, U.; de Groot, F. M. F. Ultrafast Light-Driven Electronic and Structural Changes in $LaFeO_3$ Perovskites Probed by Femtosecond X-Ray Absorption Spectroscopy. *Advanced Materials* **2025**. https://doi.org/10.1002/adma.202502932.

(11) Park, S. H.; Katoch, A.; Chae, K. H.; Gautam, S.; Miedema, P.; Cho, S. W.; Kim, M.; Wang, R.-P.; Lazemi, M.; de Groot, F.; Kwon, S. Direct and Real-Time Observation of Hole Transport Dynamics in Anatase TiO_2 Using X-Ray Free-Electron Laser. *Nat Commun* **2022**, *13* (1), 2531. https://doi.org/10.1038/s41467-022-30336-1.

(12) Katoch, A.; Park, S. H.; Jeong, K.; Lazemi, M.; Wang, R.; Ahn, H. S.; Kim, T. K.; de Groot, F. M. F.; Kwon, S. Nano-Size Effects on Decay Dynamics of Photo-Excited Polarons in CeO_2. *Advanced Optical Materials* **2024**, 2401386. https://doi.org/10.1002/adom.202401386.

(13) Miao, T. J.; Tang, J. Characterization of Charge Carrier Behavior in Photocatalysis Using Transient Absorption Spectroscopy. *The Journal of Chemical Physics* **2020**, *152* (19), 194201. https://doi.org/10.1063/5.0008537.

(14) Vura-Weis, J.; Jiang, C.-M.; Liu, C.; Gao, H.; Lucas, J. M.; de Groot, F. M. F.; Yang, P.; Alivisatos, A. P.; Leone, S. R. Femtosecond $M_{2,3}$-Edge Spectroscopy of Transition-Metal Oxides: Photoinduced Oxidation State Change in α-Fe_2O_3. *J. Phys. Chem. Lett.* **2013**, *4* (21), 3667–3671. https://doi.org/10.1021/jz401997d.

(15) *https://next-gen.materialsproject.org/materials/mp-552676*.
